# A Landscape in Transitions: Guletta, a Multiperiod Settlement along the Mazaro River in Western Sicily

**DOI:** 10.1080/00934690.2020.1734898

**Published:** 2020-03-09

**Authors:** Christopher Sevara, Roderick B. Salisbury, Michael Doneus, Erich Draganits, Ralf Totschnig, Cipriano Frazzetta, Sebastiano Tusa

**Affiliations:** a University of Vienna, Vienna, Austria; b Austrian Academy of Sciences, Vienna, Austria; c Zentralanstalt für Meteorologie und Geodynamik, Vienna, Austria; d Departimento dei Beni Culturale e dell’Identità Siciliana, Palermo, Italy

**Keywords:** landscape archaeology, remote sensing, geoarchaeology, geophysical prospection, Bronze Age, Iron Age

## Abstract

The Prospecting Boundaries project explores the Mazaro river corridor from a landscape archaeological perspective, using integrated prospection techniques to recover traces of past human activity and environmental contexts. One key research area is Guletta, a zone of dense multiperiod activity situated on the rocky plain above the river. In this paper, we detail results from recent work at Guletta, which has revealed numerous previously undocumented archaeological settlement features that appear to have been built in successive phases. Artifact analysis from corresponding surface survey indicates a mixture of locally produced and imported materials dating from the Middle Bronze to Archaic periods. Using these new results together with existing archaeological and environmental information, we present an initial interpretation of the occupation sequence of the settlement and explore the concept of Guletta as a connecting point between emerging indigenous, colonial, coastal, and interior interdependencies and interests in later pre- and protohistory.

## Introduction

The zone between coastal and interior western Sicily contains a rich matrix of archaeological resources indicative of its changing importance and functions at various periods in history. Nowhere is this in greater evidence than along the banks of the Mazaro River, a spring-fed perennial stream that flows from the hilly western interior near modern-day Salemi for more than 25 km southwest to the Mediterranean port city of Mazara del Vallo. The inland section of the river flows in a wide and gentle valley, but as it approaches Mazara del Vallo, it cuts into shallow sandstone, forming a deep-sided gorge whose rocky outcrops have provided resources and shelter for both the living and the dead for millennia. Existing information about archaeological resources in the region indicate a diversity of archaeological sites and isolated finds from the Upper Palaeolithic to the modern era and a complex spatiotemporal relationship with the river and surrounding lands (Calafato, Tusa, and Mammina 2001; Dado 2004; Di Stefano 2016; Doneus 2007; Fentress 1998; Fentress, Kennet, and Valenti 1986; Ingoglia and Tusa 2006; Mannino 1971; Mosca 2016; Nicoletti and Tusa 2012a; Tusa 1997, 1999, 2005; Tusa and Di Salvo 1988–1989). The overall impression is one of nodes of substantial activity in the landscape at various periods in time.

**Table 1.** List of principal time periods mentioned in the text.

Period	Date	Abbreviation
Neolithic	6000–3500 b.c.	
Copper Age	3500–2500 b.c.	
Early Bronze Age	2500–1500 b.c.	EBA
Middle Bronze Age	1500–1200 b.c.	MBA
Late/Final Bronze Age	1200–900 b.c.	LBA
Early Iron Age	900–734 b.c.	EIA
Colonial/Archaic	734–480 b.c.	
Indigenous Iron Age	734–550 b.c.	
Classical	480–323 b.c.	
Hellenistic	323–241 b.c.	

One of these nodes is Guletta, an area of dense multiperiod mortuary and settlement activity extending from the rocky plateau above the riverbed down to its right bank. The discovery via aerial reconnaissance of a multi-ditched structure on top of the plateau in 2003 (Doneus 2007) led to the development of the Prospecting Boundaries project. This landscape-oriented research project focused on discovering new archaeological resources through integrated archaeological prospection and analysis of diverse information sources, including geophysical prospection, geoarchaeology, airborne laser scanning (ALS), targeted and total coverage aerial photography, surface survey, and archival materials (Sevara et al. 2017, 2018, 2019, 2020). Initial evidence suggests settlement occupation from at least the Middle Bronze Age (MBA) through later prehistory. Due to the diversity and scope of its archaeological resources, Guletta has the potential to serve as a focal point for the development of our understanding of activity along the Mazaro River during later pre- and protohistory. Furthermore, it has also served as a prime location for evaluating the applicability of such integrated data collection and interpretation approaches in the specific environmental context of the western Mediterranean.

In this paper, we focus on understanding more about the development of Guletta and its position in the later pre- and protohistoric western Sicilian cultural landscape through analysis of our collected data. We first introduce the archaeological and environmental context of our project area and discuss the key aims of our research project. Next, we describe the case study area of Guletta and detail the results from the integrated prospection. Following that, we present our interpretations of the results and postulate likely developmental scenarios for the area based on the currently available information. We then situate the Guletta microregion in the wider context of known regional activity in later pre- and protohistory. Finally, in light of the new information presented here, we posit the concept of Guletta as a node of activity in a landscape of emerging cultural and socioeconomic interdependencies in Late Bronze to Iron Age western Sicily.

## The Archaeological and Environmental Contexts of Western Sicily and the Mazaro

### The Prospecting Boundaries project

**Figure 1.**
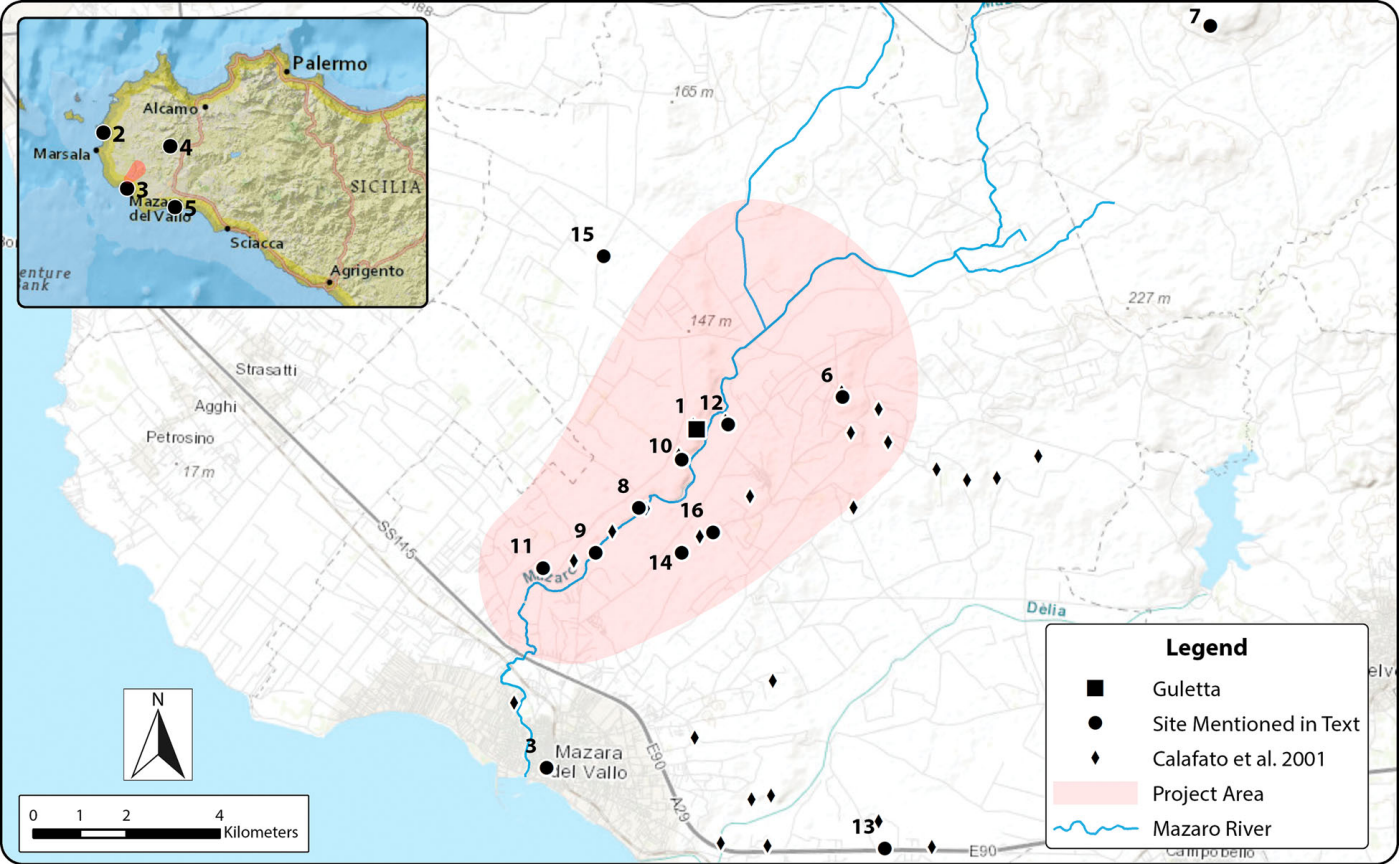
Project area, showing Guletta (1), and other sites mentioned in the text. 1. Guletta, 2. Motya, 3. Mazara, 4. Salemi, 5. Selinous/Selinunte, 6. Roccazzo, 7. Mokarta, 8. Castelluccio di Mazara, 9. Archi, 10. Gattolo, 11. San Miceli, 12. Grotte Portazza, 13. San Nicola, 14. Contrada Spadaro, 15. Contrada Mirabile, and 16. Granatelli. Background image © ESRI.

Work at Guletta is a part of the Prospecting Boundaries project, which covers roughly 70 km^2^ along both sides of the Mazaro River (Figure 1). Ancient historians mention the Mazaro (historically Mazaros or Mazarus, Greek Μάζαρος) as a natural boundary in the west in the later part of the Iron Age (Diodorus xi.86.2; Thucydides 6.6). Mazara and the Mazaro River appear on maps of Sicily from as early as the 11th–12th century, such as those in the Medieval Islamic Book of Curiosities (Rapoport and Savage-Smith 2014, 138fol. 32b–32a Book 2, Ch. 12), indicating the historical importance of the river as a landmark, resource, and component of the cultural landscape. While Prospecting Boundaries is concerned with human activity in the region during all periods, of special interest is the concept of the Mazaro region as a zone of interaction and shifting sociocultural interests from the end of the 2nd through the mid-1st millennium b.c. Prior archaeological research has identified discrete areas of human activity from several periods along the Mazaro. What is less well understood is how life and land use in the transition zone between coast and interior may have changed as increasing population density, new cultural groups, strain on local ecosystems, and subsistence requirements began to iteratively affect the sociocultural landscape of western Sicily.

### Western Sicily in later prehistory

Despite extensive work in a few key areas, relatively little is known about much of the Bronze Age in western Sicily in comparison to other parts of the island. Currently, it appears that the distinctive cultures of the Early Bronze Age became more uniform by the middle of the 2nd millennium b.c. (Tusa 1999, 460). During the MBA, traditional models indicate that cultural development seems to have been influenced by connections with Aegean trading networks and contacts throughout the Mediterranean (Leighton 1999, 147; Kolb 2007, 176; Russell 2017, 66), though perhaps not to the same extent as at sites in other parts of Sicily. In the western part of the island, survey results indicate a range of MBA sites in a variety of inland locations, pointing to a period of settlement expansion (Leighton 2005, 272). However, the relative paucity of imported and import-derived goods at many MBA sites makes it difficult to determine the extent to which populations in the western part of Sicily derived significant direct economic or cultural influence from Aegean contact networks (Blake 2008; Russell 2011, 138; Russell 2017; Spatafora and Sciortino 2015). Toward the end of the MBA, it appears that some local populations aggregated to larger, more easily defensible inland areas (Bietti Sestieri 2015, 90; Kolb 2007, 177; Leighton 1999, 192; Nicoletti and Tusa 2012a; Tusa 1999; 2009, 28). By the Late Bronze Age (LBA), current thinking is that three distinct cultural groups emerged: the coastal tradition derived directly from the Thapsos-Milazzese culture, the related Mokarta group connected to Pantalica Nord and Cassibile, and the continental Ausonian (Nicoletti and Tusa 2012a, 116).

There is currently little archaeological evidence in the west to help understand the transition between the end of the Bronze Age, characterized by the abandonment of sites such as Mokarta, a prominent LBA village in the western interior near modern-day Salemi (Kolb 2007, 176; Mannino and Spatafora 1995; Nicoletti and Tusa 2012b; Sevara et al. 2020; Spatafora and Mannino 1992; Tusa 1999, 625; Tusa and Nicoletti 2000), and the Early Iron Age (EIA). However, it seems that by ca. 800 b.c., a group of people whose origins are the subject of debate had established a complex societal structure distinct from that of preceding periods. This structure was characterized by the development of strategically placed large nucleated hilltop settlements (as discussed by e.g. Ferrer 2016, 903; Kolb 2007, 177; Leighton 1999, 227; Tusa 1999). Thus, a fully established indigenous population in the west had already developed a distinctive social and material culture by the time of the Phoenician settlement of Motya and subsequent establishment of a Greek colony at Selinous. Archaeological and historical records indicate that by the middle of the 1st millennium b.c., these three main cultural groups were enmeshed in a fuzzy web of multidirectional contact, trade, and cultural transmission that included cohabitation, changes in burial practice, adaptation of pottery styles, adoption of religious practices, and periodic conflict (Balco 2012; De Angelis 2003a, 2003b; Hodos 2006, 115, 129).

### Archaeology along the Mazaro

Other parts of western Sicily have been the subjects of more systematic field survey in recent years (e.g. Blake and Schon 2010; Kolb 2007; Spanò Giammellaro, Spatafora, and van Dommelen 2008). However, published information about many of the sites around the Mazaro is limited to an inventory of mortuary and settlement sites found via extensive survey, serendipitous events, and local informants (Calafato, Tusa, and Mammina 2001; Doneus 2007; Di Stefano 2016). This includes a report of a significant Neolithic settlement at Castelluccio di Mazara, on the left bank of the river in the center of the project area (Calafato, Tusa, and Mammina 2001, 37; Mannino 1971, 41; Tusa 1999, 212). Documented Copper Age activity is clustered to the northeast, near the well-known site of Roccazzo (Calafato, Tusa, and Mammina 2001, 44; Tusa 1997; 1999, 290–294). Aside from indications of Bronze Age settlement activity at Contrada Archi, related to the Mokarta group (Nicoletti and Tusa 2012a), evidence from the Early to Middle Bronze Age comes mainly in the form of largely empty or disturbed grave structures, such as those at Gattolo, Granatelli, and Grotte-Portazza (Calafato, Tusa, and Mammina 2001, 38; Ingoglia and Tusa 2006; Tusa 1999, 441) (Figure 1). Furthermore, the only cartographic indication of prehistoric archaeological activity is the toponym *grotta* (cave) on a 1:50.000 series Istituto Geografico Militare (IGM) topographic map from 1896, likely indicating one of the more prominent later period funerary structures dug into the side of the gorge (IGM 1896).

Later period activity is present on both sides of the river. In the 1980s, surveys to the northwest of the Mazaro near Contrada Mirabile revealed numerous potential habitation sites and associated material from the 4th century b.c. through the Roman period (Fentress, Kennet, and Valenti 1986; Fentress 1998). In a recent intensive survey, Mosca (2016) identified extensive settlement activity dating from the Hellenistic period to the 6th–7th century a.d. near San Miceli and used this to argue for the development of a possible urban center or village there in early history (Mosca 2016, 9). Greek/Punic period activity seems to have increased in the area between the Mazaro and the Delia rivers, as evidenced by settlement and mortuary features at San Nicola and Contrada Spadaro (Calafato, Tusa, and Mammina 2001, 14; Di Stefano 2016, 36) and numerous surface finds scattered in the area, some of which may indicate significant settlements. From the beginning of the Roman period, the number of recorded settlement, mortuary*,* and associated materials increases significantly on both sides of the river. The remains of many of their successors, in the form of Arab period *rahals* (villages) and later rural central fortified farmhouses (Dado 2004; Mosca 2016, 6–7; Samuels 2012, 48; 2017, 92; Schneider and Schneider 1976, 67), often stand in similar locations to their early historic counterparts (Dado 2004; Rotolo and Civantos 2013; cf. Johns 1992, 416).

### Environmental context and modern land use

The bedrock of the project area mainly comprises Quaternary to Pliocene sediments. The elevated areas on both sides of the Mazaro River consist of Early Pleistocene marine calcareous sandstone up to several tens of meters thick, forming a prominent northwest-southeast trending calcarenite plateau. Below the calcareous sandstone are slightly folded Pliocene marl and claystone overlying upper Tortonian clastic sediments (D’Angelo and Vernuccio 1992). This sandstone is a suitable building material that has been extensively exploited. The contact between the Pleistocene calcareous sandstone and the Miocene sediments below is exposed in the lower part of the slope below Guletta. The Early Pleistocene sandstone is covered in a few places, e.g. at the nearby settlements of Roccazzo and Montagna della Metá, by several meters of fine-grained, well-sorted Pleistocene eolianite sandstone. Considerable Holocene sediments are found directly at the coast or in lower areas of the Mazaro River (D’Angelo and Vernuccio 1992).

Macro-regional pollen and charcoal data suggest the presence of forests in western Sicily during the Neolithic and Copper Ages, with a sharp increase in weeds, crops, and herbaceous plants and a corresponding decline in tree species beginning ca. the 8th century b.c. (Calò et al. 2012; Stika, Heiss, and Zach 2008; Tinner et al. 2009). The current environment of the upland plain onto the Early Pleistocene calcareous sandstone is a Mediterranean pseudosteppe with arid, rocky outcrops and low scrub vegetation, which is known locally as *sciara* (pl. *sciare*). Soils that are thicker and more fertile are found in areas with Pliocene and Holocene sediments. In terms of climate, the region today is characterized by a Mediterranean climate of dry summers with mean annual temperatures of 18°C and a mean annual precipitation of less than 600 mm (Gini and Misuraca 2009).

**Figure 2.**
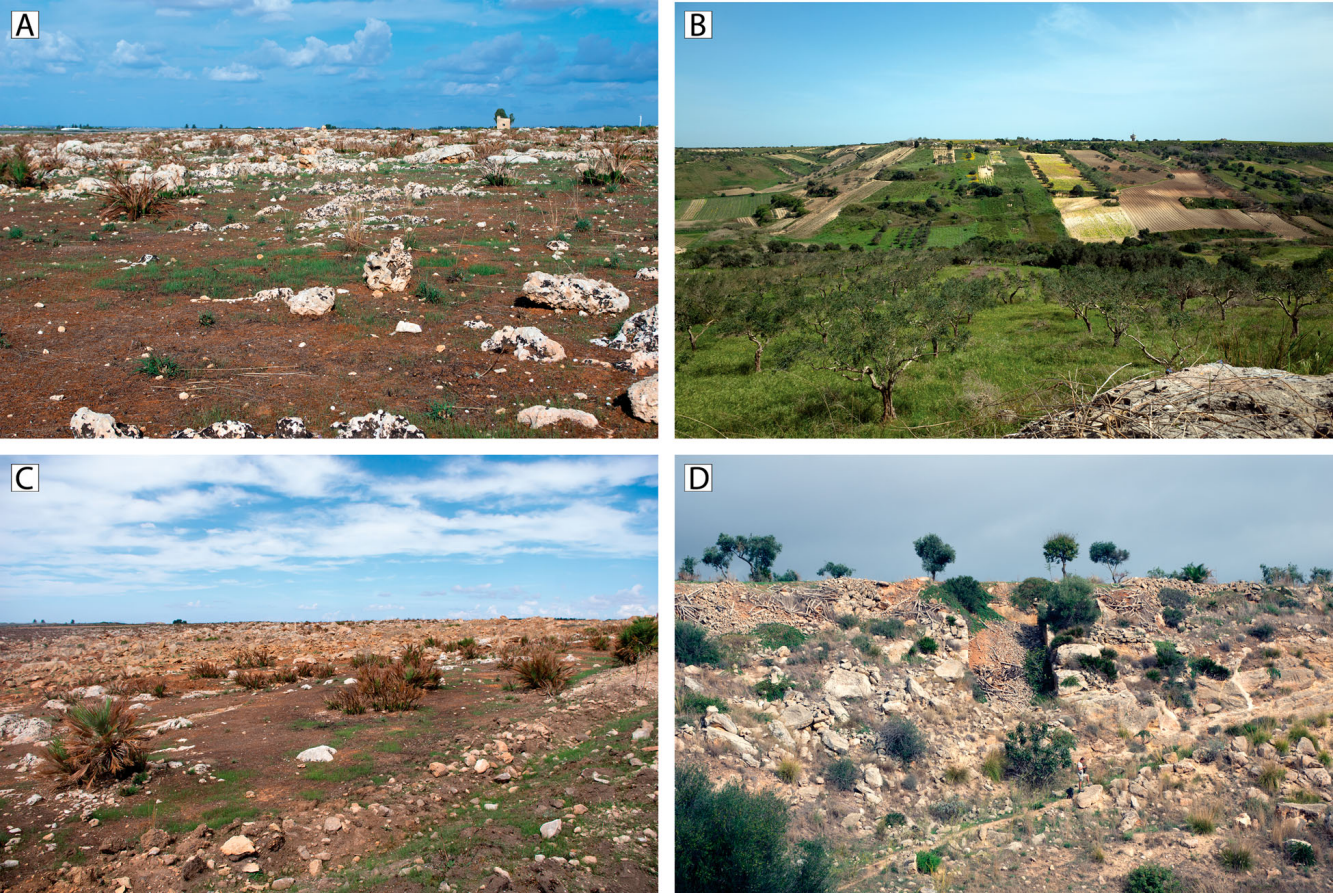
Modern land use and environmental contexts within the project area. A) Sciara di Mazara del Vallo, facing north. B) Mixed agricultural contexts and land fragmentation in the Mazaro River Valley, facing south. C) Soil recovery, a process of bedrock pulverization for rapid creation of arable land, facing west. D) Quarrying and infilling of soil for creation of agricultural surfaces, facing northwest. Photographs: C. Sevara.

Modern land use in the region has had a dramatic impact on surface visibility and preservation of the archaeological record. Land use in our survey area varies: the sciare are largely given over to pasturage and quarrying, with the latter apparently a common practice in the region from antiquity to modern times (Lombardo 2003). The southern and northern parts of the project area are dedicated to agricultural production, including cereal crops, vineyards, and olive and fruit trees. Recently, a process of accelerated soil creation, with large areas of the sciare being pulverized with heavy machinery to create arable soil from the bedrock substrate (e.g. Fantappiè, Priori, and Constantini 2015, 332), has radically transformed large portions of the plateau and threatens to subsume significant parts of the current remains of the natural and cultural landscape (Gini and Misuraca 2009, 125). Large-scale soil deposition designed to infill large areas for agricultural use has capped the original ground surface in many areas (Sevara et al. 2018). Land fragmentation, a visible relic of historical systems of land management and inheritance, further reduces surface visibility (Figure 2). Along with a switch to more intensive agricultural practices in the last half of the 20th century, these different types of land use have varying impacts on the visibility and preservation of archaeological resources (Fentress 1998; Mosca 2016, 7; Ingoglia and Tusa 2006, 537; Sevara et al. 2018), affording us pockets of relative historic continuity amidst greater areas of significant modern change.

## The Guletta Case Study: Methodology and Data Acquisition

**Figure 3.**
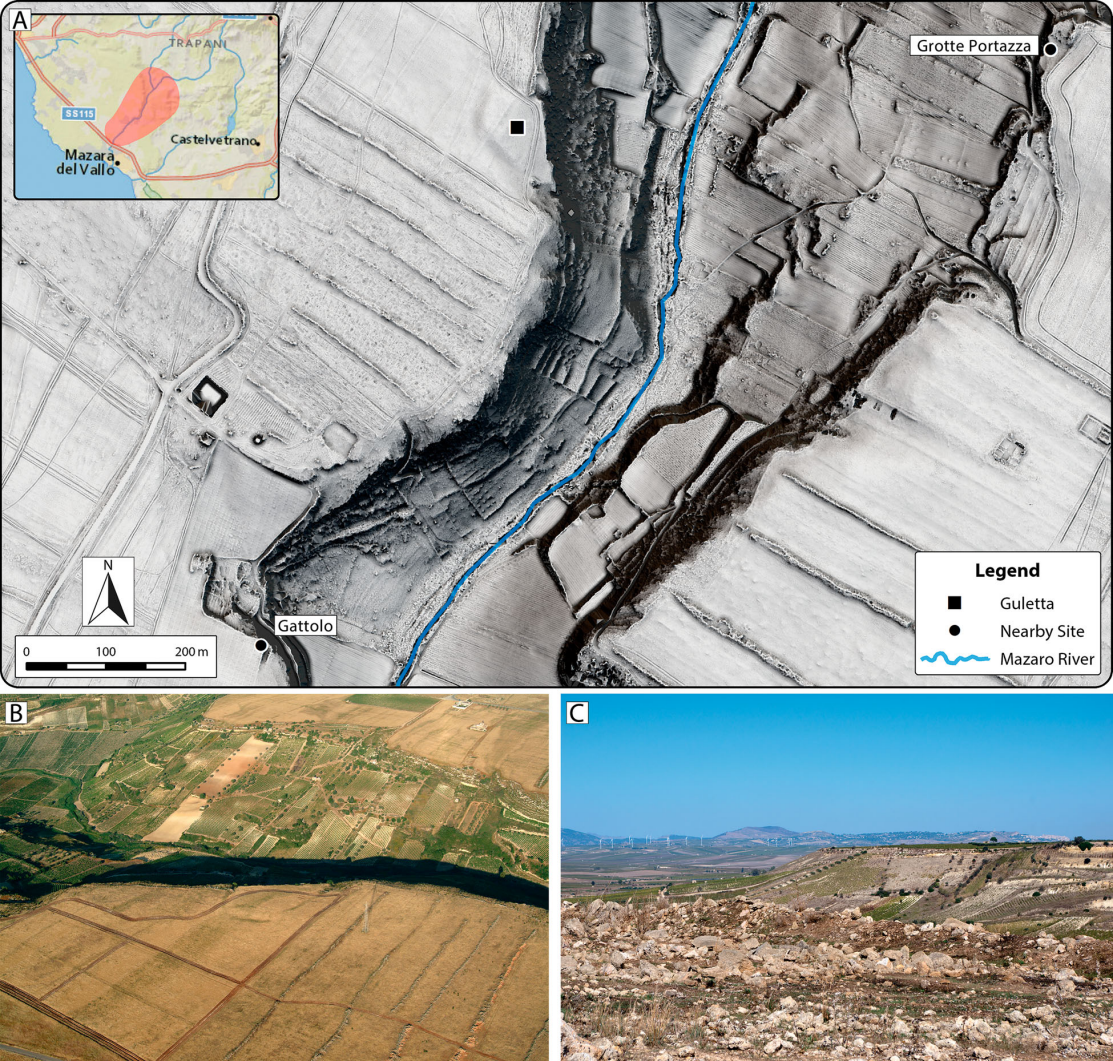
A) 50 cm spatial resolution ALS-derived DTM visualization (Sky-view factor over multiple hillshade) depicting terrain in the vicinity of Guletta and proximity of Guletta to other identified sites in the area. B) Aerial photograph of Guletta, from 2003. Image source: University of Vienna Aerial Archive. C) View of interior from Guletta. Locations of modern towns and prehistoric mountain top settlements, including Salemi and Monte Polizzo, can be seen clearly from Guletta’s location. Photographs: C. Sevara.

The design of Prospecting Boundaries arose from the need to understand developments along the Mazaro River at multiple scales. Discoveries from aerial surveys conducted in the early 2000s (Doneus 2007) and evidence present in historical aerial imagery indicated that there was much left to discover in the region along the Mazaro and that a suite of archaeological prospection methodologies could provide data for a fuller understanding of the nature and context of past human activity in the region. Guletta, along with its surrounding area, is one of these discoveries and has become a focal point of this research. The central portion of the site occupies a topographically prominent area at the edge of the Mazaro gorge and close to the inland edge of the sciara that affords an extensive view of the region, including nearby mortuary and settlement sites, prominent coastal areas, and sites in the western interior (Figure 3). Although prominent, prior archaeological investigation in the region failed to identify it, even though significant mortuary and settlement sites from other periods had been documented nearby.

A site visit to the structure that was identified at Guletta during the 2003 aerial photography campaign carried out by the University of Vienna Aerial Archive (Doneus 2007, 281) confirmed the presence of multiple ditches. Moreover, a number of locally produced ceramics of pre/protohistoric origin were discovered in the vicinity. Initial archival research revealed that traces of the ditched structure and a surrounding enclosure appear in orthoimagery from as early as 1941. Although located in an area of high visibility and defensibility, a particular characteristic of a number of known MBA sites in Sicily (Doonan 2001, 173), it is not in a place that would traditionally be considered typical for an indigenous Iron Age settlement. In fact, prior to 2003, the area around Guletta had been known chiefly for its Roman-era settlement component (north of the current study area), exposed quarries of historic and possibly prehistoric origin, and the presence of prehistoric and historic rock-cut tombs along the cliff face overlooking the river (Calafato, Tusa, and Mammina 2001, 39; Di Stefano 2016, 50; Tusa 1999).

### Integrated prospection at Guletta

A bespoke orthorectified aerial photography and ALS data collection campaign was conducted over the entire project area to obtain information about the physical environment and exposed earthworks and features (see Sevara et al. 2018 and Supplemental Material 1 for ALS survey details). Archaeological terrain models were calculated from ALS point clouds using filtering algorithms specifically designed to preserve traces of archaeological features in the terrain while removing vegetation (Doneus and Briese 2006, 2011; Doneus et al. 2008). Radiometrically corrected reflectance maps simulating infrared orthoimagery were calculated from the amplitude data present in the ALS point cloud (Sevara et al. 2019), and visualizations highlighting topographic discontinuities were calculated from the surface models using the software packages OPALS and RVT (Doneus 2013; Kokalj and Hesse 2017; Mandlburger et al. 2009). Historic aerial imagery and topographic maps, acquired from the IGM, provided key information about the landscape, land use, and cultural history of the region at multiple points in time. All of these materials were collected, processed, and integrated for analysis in a GIS environment, together with existing information about archaeological resources in the region (see Sevara et al. 2018, 2019 for further details).

Areas for geophysical prospection, intensive surface survey, and geoarchaeological survey were delimited based on an initial interpretation of the historic and modern remote sensing data. Geophysical prospection at Guletta was carried out by Archeo Prospections® of the Zentralanstalt für Meteorologie und Geodynamik (ZAMG) in the spring of 2016. Roughly 9 ha of magnetic data was collected across the central portion of the settlement (see Supplemental Material 2 for equipment details). Using the magnetometry results as a guide, six areas of interest totaling 2.5 ha within the survey area were chosen for the GPR survey. Surface survey was conducted in early April 2016 by members of the Prima Archeologia (PA). An area corresponding roughly to the area covered by the geophysical prospection was divided into 28 units of 100 × 100 m, from which artifacts were collected, yielding over 300 ceramic, metal, and stone objects. Diagnostic artifacts were point-located using a handheld GNSS device. Ground surface visibility was variable, with most of the site being covered by patchy low grasses and scrub vegetation. Results from these surveys, detailed below, were then combined with the existing cartographic and remote sensing datasets for further interpretation. The resulting dataset represents several different scales and resolutions of information (see Supplemental Material 3 for dataset details, and see Sevara et al. 2018, 2019 for technical details and processing specifics for datasets).

## The Guletta Case Study: Prospection Results

### Aerial photography and airborne laser scanning

**Figure 4.**
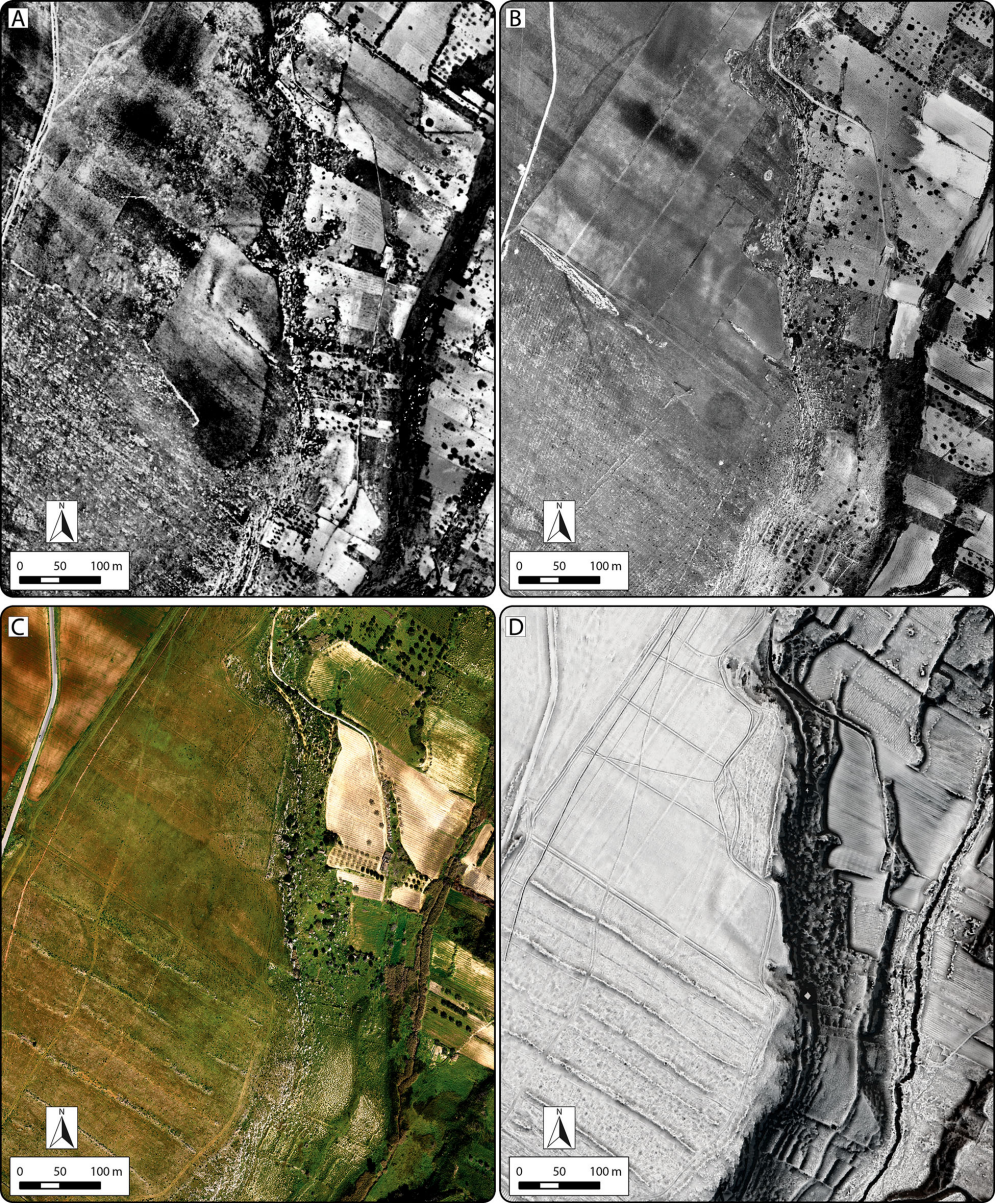
Images of Guletta from 1941–2016, showing different land use. Note land consolidation and significant clearance between 1975 and 2016. A) Aerial photograph from April 1941. B) Aerial photograph from May 1975. C) Aerial photograph from February 2016. D) 50 cm spatial resolution ALS-derived DTM visualization (Sky-view factor, local relief model and multiple hillshade) from data collected in February 2016. Images A and B: Istituto Geografico Militare, v1941-f254-4-108, v1975-f257-VIII-77, used with permission, authorization #7050.

**Figure 5.**
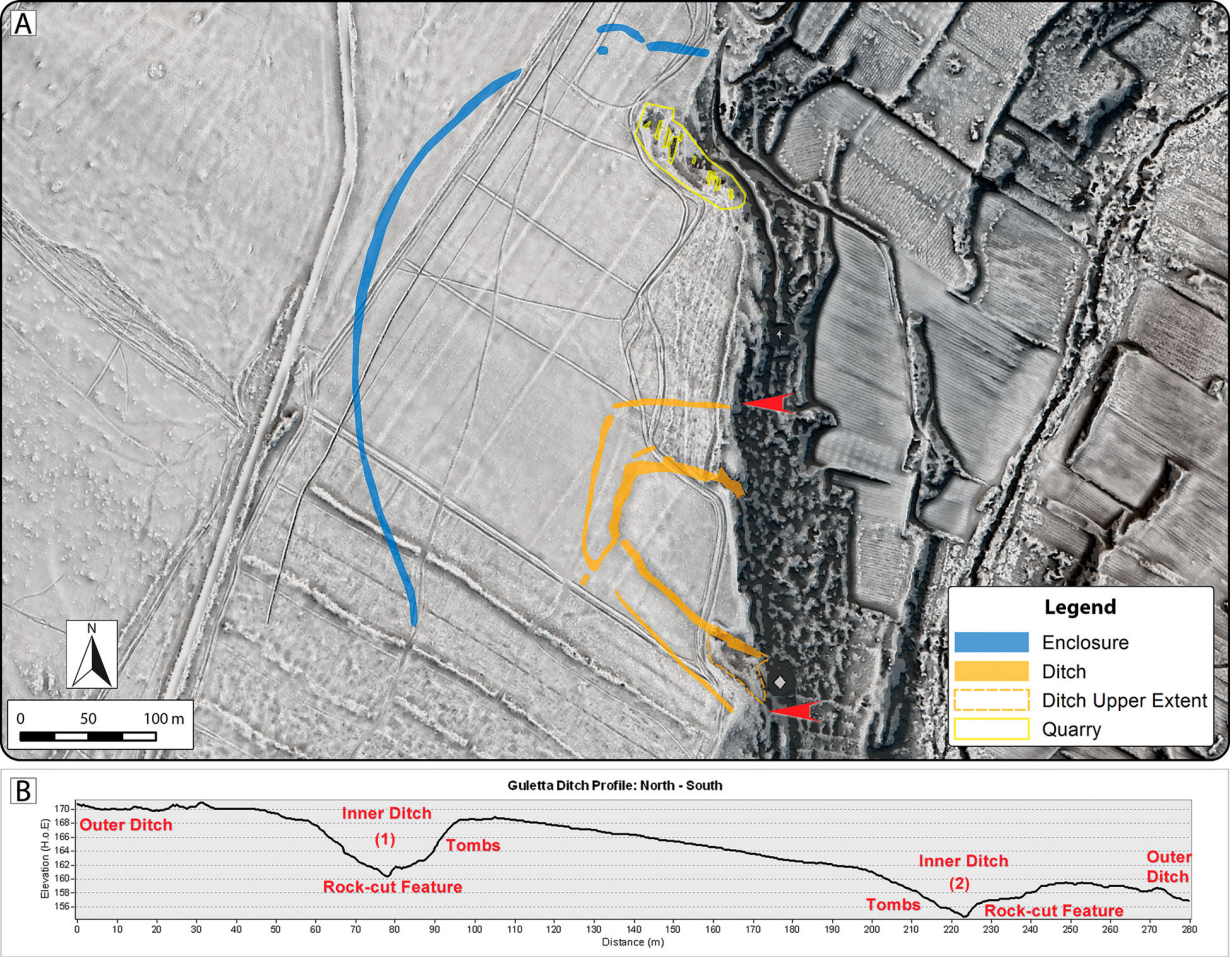
A) Composite interpretation of pre/protohistoric features at Guletta visible in remote sensing data from 1941–2016 over a 50 cm spatial resolution ALS-derived DTM visualization (Sky-view factor, local relief model and multiple hillshade). Red arrows indicate profile extents. B) Profile of exposed ditches at edge of gorge.

Examination of visualizations and surface models derived from ALS data, together with reflectance maps and aerial imagery, provided detailed insight into the extent and complexity of landscape change and the visible features and structures at Guletta. Superpositioning of relict field systems, terracing, transport routes, and quarry areas can be seen on the plain surrounding Guletta, as well as the terraces that stretch down to the river. The aerial imagery has also proven invaluable for understanding historic land use changes and the effect these changes have on the visibility of archaeological resources. In particular, significant modification of the land surface by clearance for vineyards and other agriculture is particularly well documented in images from the 1940s–1970s, while terraces along the banks appear to have already been in place (Figure 4). Soil and vegetation marks indicate portions of archaeological features that have since largely disappeared from view. Data from these images are also useful for tracking soil movement, the growth of quarries, and large-scale modification of the landscape (Sevara et al. 2018). For example, at Guletta, we can see that portions of some features may have been exposed as late as the 1970s and then subsequently infilled, likely during the ensuing clearance of the vineyard remnants and plowing of the numerous firebreaks that criss-cross the site area (Figure 4B–C). Together, the composite interpretation of the visualizations, aerial imagery, and surface model profiles clearly depict the exposed areas of the inner ditch surrounding the interior portion of the site and capture the rock cut edges of the ditch structures and quarries visible in the area (Figure 5).

### Magnetometry

**Figure 6.**
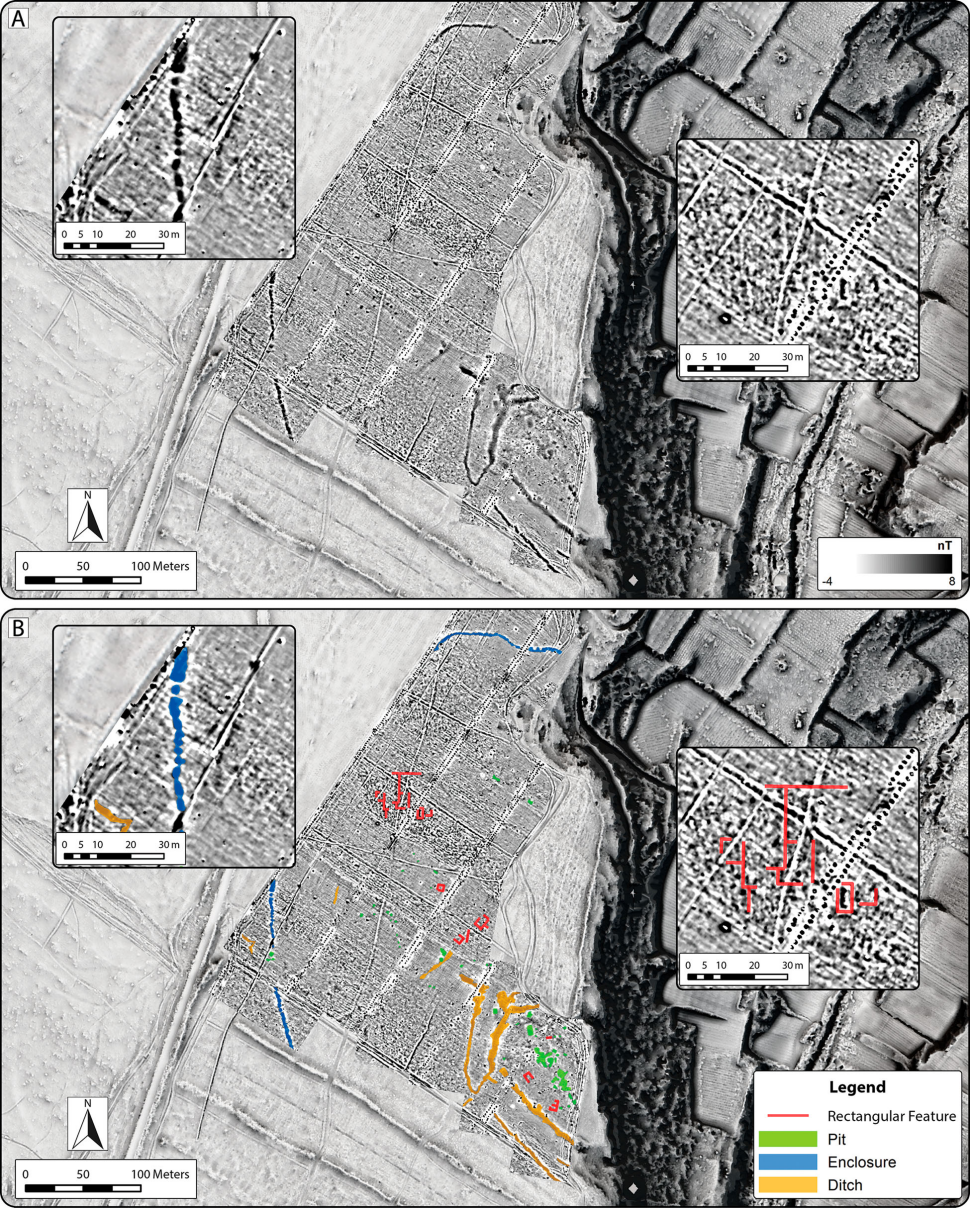
Results of magnetometry survey at Guletta. A) Magnetogram depicting features with high magnetic contrast to surrounding environment. B) Interpretation of principal pre/protohistoric features visible in the magnetic prospection dataset. Insets: Section of outermost linear anomaly (left) and area of potential rectilinear feature (right). Background: 50 cm spatial resolution ALS-derived DTM visualization (Sky-view factor, local relief model and multiple hillshade).

Data from the magnetometry survey indicates potential archaeological features from several discrete phases of activity at Guletta (Figure 6). What appear to be most recent are large linear anomalies corresponding to areas repeatedly plowed for firebreaks, visible at numerous points throughout the other modern remote sensing datasets. Additionally, a series of five extremely high contrast NE–SW running linear alignments of paired circular anomalies can be seen crossing the survey area at evenly spaced intervals of roughly 65 m. These appear to interrupt other anomalies visible in the data. Closely spaced, narrow linear features lie at right angles to these alignments and are likely associated with them. Examination of historic aerial imagery (Figure 4B), together with material found in situ, indicates that these features are most likely associated with a vineyard created and abandoned during the mid–late 20th century.

A series of well-defined linear anomalies enclosing an area of approximately 109 × 160 m are visible in the southeastern section of the surveyed area. These anomalies correspond to the location of the ditched structure visible in the aerial and ALS data and appear to consist of at least two parallel ditches covering a distance of between 18–26 m. The ditched structure appears to have a large south-facing opening whose eastern edge may have been interrupted by the subsequent vineyard activity. A linear feature along the western edge of the presumed entryway appears to connect the inner and outer ditches. Additionally, numerous rectangular and sub-rectangular anomalies of varying sizes along the edges of the innermost ditch (Figure 6B) are in some cases superimposed upon other possible features in addition to the ditch, and are difficult to separate. Uneven, rocky terrain conditions made it impossible to survey the northern segments of this area, but corresponding aerial imagery indicates subsurface linear features manifesting as crop and soil marks running to the Mazaro gorge, where corresponding ALS-derived datasets clearly depict exposed sections of the ditches in relief (Figure 5).

In addition to the ditched enclosure, sections of two linear features can be seen at the northern and southwestern edges of the survey area. These are likely sections of a single linear feature surrounding the ditched structure, other components of which can be seen in aerial imagery (Figure 6, left insets). From the magnetometry data, it appears that the feature may have been constructed as a series of pits. If considered together as one feature, this would have enclosed an area of ca. 13 ha around the double-ditched structure described above. A gap in the southwestern section of the anomaly may have served as an entrance. A further cluster of pits, varying in size from 1.4–3.5 m in width, have been identified in the south-central section of the surveyed area, just outside the double-ditched structure, but still within the bounds of the outer enclosure.

Finally, additional faint linear negative anomalies can be seen in the central section of the survey area (Figure 6, right insets). When grouped together, these could form sections of a large rectangular building of approximately 40 × 48 m. At least three separate rooms can be identified within this building, and smaller rectangular features can be seen nearby. Additionally, the complex does not appear to align with any current or historic agricultural features. It is difficult to say whether these features are contemporary, although some appear to be connected and similarly aligned.

### Ground penetrating radar

**Figure 7.**
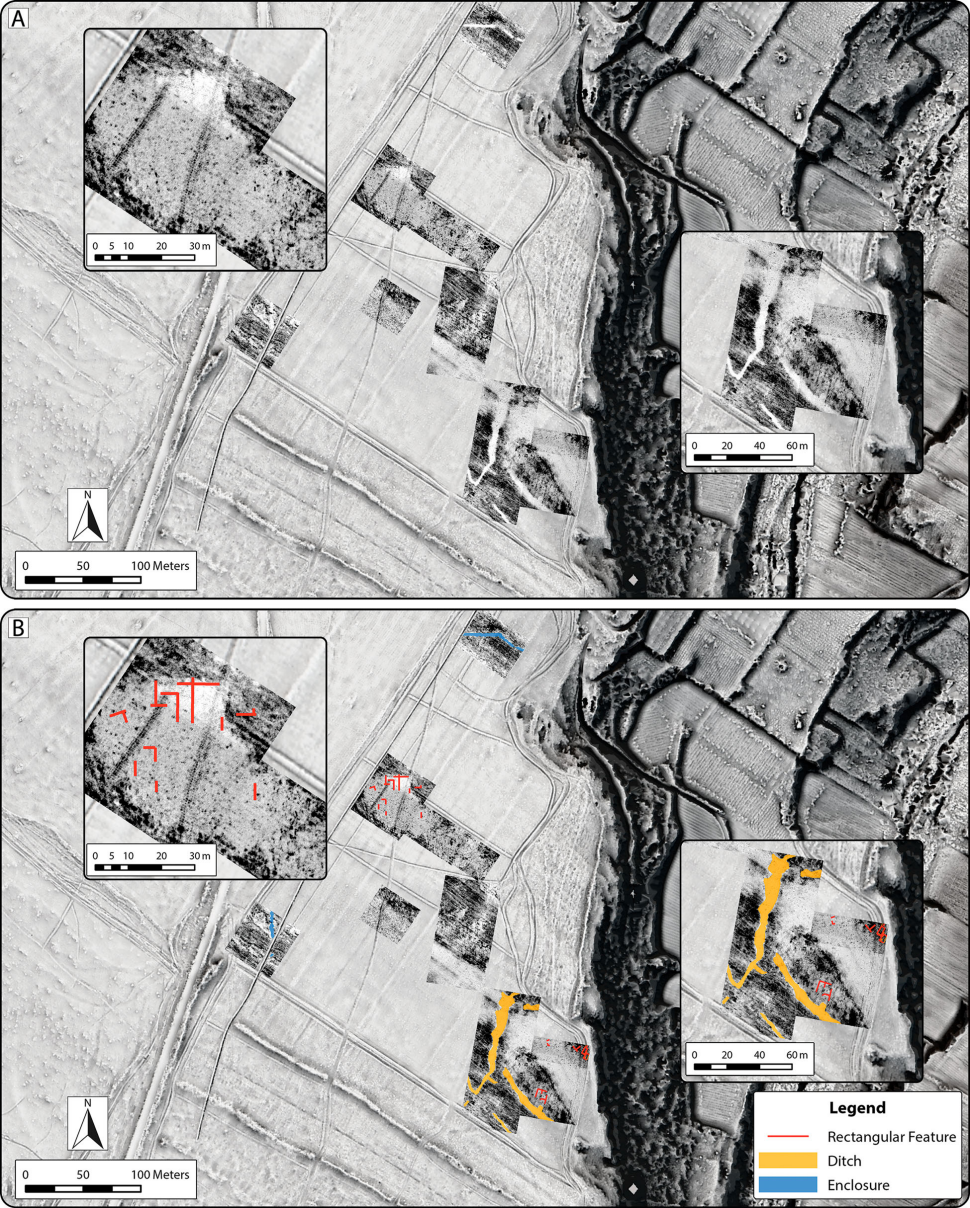
Results of GPR survey at Guletta, showing a composite depth slice at 1–1.5 m below modern ground surface. A) Radargram depicting features. B) Interpretation of principal pre/protohistoric features visible in the GPR dataset. Insets: Area of potential rectangular feature (left) and area of inner ditches where inner and outer ditches connect (right). Background: 50 cm spatial resolution ALS-derived DTM visualization (Sky-view factor, local relief model and multiple hillshade).

In the area of the double ditch, GPR results provide depth data and indicate outlines similar to those seen in the magnetometry data for both the inner and outer ditches. Surveyed sections of the inner and outer ditches are estimated to extend to depths greater than 1.5 m below the modern ground surface (Figure 7). In the northeastern section of the area, several rectangular structures were identified near the cliff edge. These are similar in size and shape to exposed quarry pits on other parts of the site. In the southern part of the survey area, just north of the inner ditch, a ca. 10 × 10 m wide area consisting of parallel linear features may also indicate a building. Unfortunately, the radar data does not clarify the superpositioning of features within and around the inner ditch.

GPR data from the area where the magnetometry survey indicates a potential large rectilinear/rectangular multi-room structure provided some information that appears to corroborate the interpretations of the magnetometry data (Figure 7, left insets). A roughly rectangular area where the sediments are more heavily compacted compared to the surroundings was identified within the magnetic anomalies. Furthermore, linear features were identified along similar alignments to those identified in the magnetometry data (Figure 6, right insets). These indicators are extremely ephemeral, possibly because of low contrast between the wall construction material and the surrounding bedrock or poor subsurface preservation in this area. Therefore, what can be seen in the prospection data are likely the remnants of building foundations or sub-foundations.

### Surface survey

Material recovered from the Guletta survey currently indicates activity over ca. 1,000 years, spanning the 15th–5th centuries b.c. A total of 305 pieces of material were recovered from the surface. Overall, 91% of the surface ceramics can be correlated to locally produced ceramics from the indigenous Iron Age and the corresponding Archaic-Classical period. Diagnostic material from the indigenous Iron Age is present in most of the 28 collection units and includes a large amount of coarseware storage vessel fragments similar to those found at other indigenous areas of activity in the region. Fragments of imported Greek and Punic wares, including so-called Ionian B2 cup fragments, were also found at both the northeastern and southwestern ends of the collection area. Material from the MBA (6%) and small quantities of EIA material (3%) were also recovered. Curiously, artifacts from later periods (excluding modern debris associated with the former vineyard activity) seem to be entirely absent, although earlier surveys have noted significant amounts of Roman material ca. 300 m to the north of the current survey area (Calafato, Tusa, and Mammina 2001, 39).

Several factors influence results from the surface survey, including variable surface visibility, significant post-depositional disturbance associated with transhumance and agriculture, and localized, repeated plowing to establish and maintain firebreaks. Regularly plowed modern firebreaks yielded high amounts of material. Outside of the firebreak areas, many of the artifacts collected were weathered, indicating that they had been exposed for some time. The distribution and visibility of materials on the surface also seems to be influenced by the historical use of the land as a vineyard. Nevertheless, unlike other parts of the Prospecting Boundaries study area, most of the section of Guletta covered by this survey has not been subjected to deep plowing, pulverization of the bedrock, or significant deposition of transported overburden. Therefore, although the specific spatial context of the material and its recovery have been influenced by post-depositional factors, the quantity and relatively homogeneous nature of the material can be taken as general indicators of time period and prior activities.

### Geoarchaeological survey

Geoarchaeological reconnaissance survey and examination of historic imagery, geological maps, and ALS data for the upland plain around Guletta support interpretations of extensive impacts from plowing, pulverization of the bedrock to accelerate soil formation, and deposition of sediments transported from elsewhere on the island. Soils on this plain are reddish-brown and coarse, similar in appearance to *terra rossa*, and suggest that significant soil development has not occurred in the region in recent years. Despite this, older evidence of plowing around small farmsteads and small sediment traps in rocky outcrops on the sciara suggest that the plain may have been subject to deflation in conjunction with ongoing environmental change. Average slope on the plateau is less than two degrees, suggesting that soil loss via surface run-off is highly unlikely (see also Fantappiè, Priori, and Constantini 2015). Therefore, assuming a pattern of deforestation and vegetation change similar to that suggested for other parts of western Sicily during the latter part of the mid-1st millennium b.c. (Heinzel and Kolb 2011, 104; Stika, Heiss, and Zach 2008, s147; Tinner et al. 2009), the upland plain around Guletta may have become increasingly more conducive to pasturage and grazing during that time.

Terraces and exposed profiles within the gorge show a quite different composition. The presence of dark greyish-brown and brown loamy soils in the terraces, under walls, and under terraformed overburden contrasts sharply with the coarse, reddish sediments on the sciara. The obvious interpretation is that more fertile soils could be found within the gorge, from prehistory until today, and have been protected by terracing. It is also possible that the sciara itself was more fertile in the past, and that soil has been lost to erosion with the advent of land clearance and modern agricultural practices.

### Prospection data and artifact distribution

**Figure 8.**
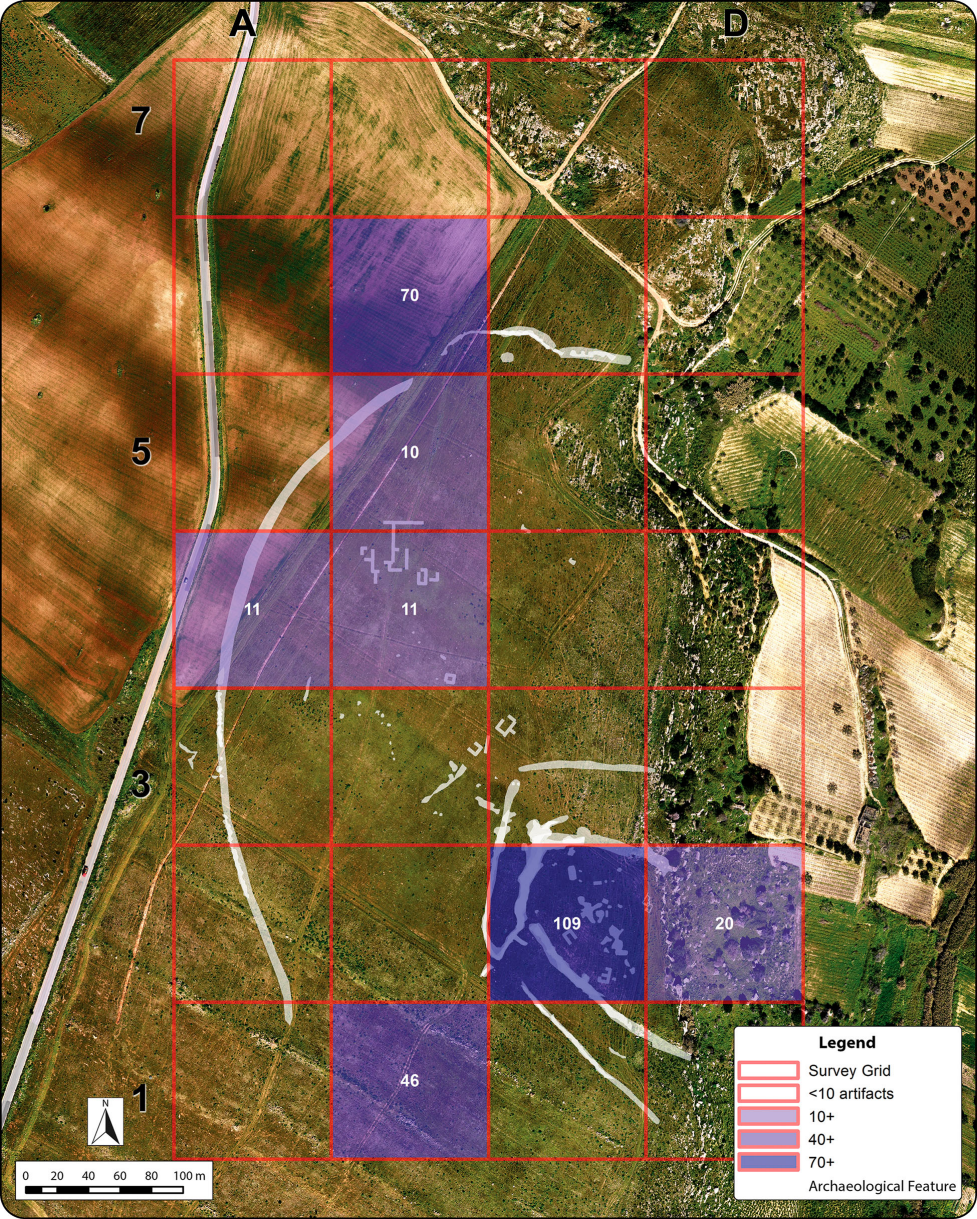
Distribution of artifacts collected during intensive survey, superimposed over identified archaeological features. Significant concentrations correspond to principal features located during interpretation of remote sensing, magnetometry, and GPR data. Numbers in squares indicate artifact counts. Background: orthoimage from February 2016, depicting ground conditions near time of survey.

Together, the prospection and survey data collected at Guletta have provided critical insight into the structural composition of the site and its surroundings. Components of many features can be observed in multiple datasets, while some datasets provide unique insights into structures and features at the site. In particular, analysis of materials recovered from the surface survey currently provides our strongest link to the timeframe of activities in the area, and it is worth noting that the bulk of the surface finds were recovered from three main loci around features indicated in the prospection data (Figure 8). The largest quantity of material recovered from a single unit comes from within the multi-ditched enclosure, where roughly 80% of the material collected can be dated to the indigenous Iron Age and Archaic/Classical periods and includes ceramics identifiable as Greek and Punic imports. A further 15% of the recovered material can be dated to the MBA, which is a significant increase over MBA material recovered from the remainder of the survey area. A significant amount of material was also present near the large rectilinear geophysical anomaly. In the northeastern part of the survey area, a third concentration located near a section of the outer enclosure feature also yielded a comparatively high amount of material, with diagnostic materials dating to the EIA and indigenous Iron Age/Archaic/Classical periods*,* including both Punic and Greek imported material*.* Given the pattern and density of the materials recovered, it seems likely that their distribution is largely an effect of local landscape modification processes (e.g. from historic land use modification, such as vineyard planting and firebreak plowing) and is not related to longer distance soil transportation, redeposition or manuring. Thus, although variable surface visibility and subsequent land use modification may be affecting the recovery of artifacts, we can nevertheless be reasonably certain that the artifacts recovered have not been transported a significant distance since their initial deposition.

## Interpreting the Development of Guletta

What is emerging from the analysis of data gathered at Guletta is evidence of a complex cycle of activities in the area between the mid-2nd and mid-1st millennia b.c. Above all else, it seems likely that the central portion of the area surveyed contained a pre- and protohistoric indigenous settlement of moderate size, with an occupation sequence of at least 1000 years. Materials recovered from the site are indicative of domestic activity and trade, linking Guletta with regional activities and settlements on the coast and in the interior. The superpositioning and distinctive alignments of various features also indicate that they may belong to distinct phases of construction. The dissimilar nature of the inner and outer ditch and possible remodelling further support the interpretation of different construction phases and spans of use. Additionally, the cross-cutting of several exposed features at Guletta, particularly the mortuary features along the sides of the inner ditch and the ditch construction evidence from the GPR data, provide additional support for multiple periods of occupation at Guletta. It seems clear that people were occupying and reusing this space, if not continuously, then at least repeatedly over a long period of time.

**Figure 9.**
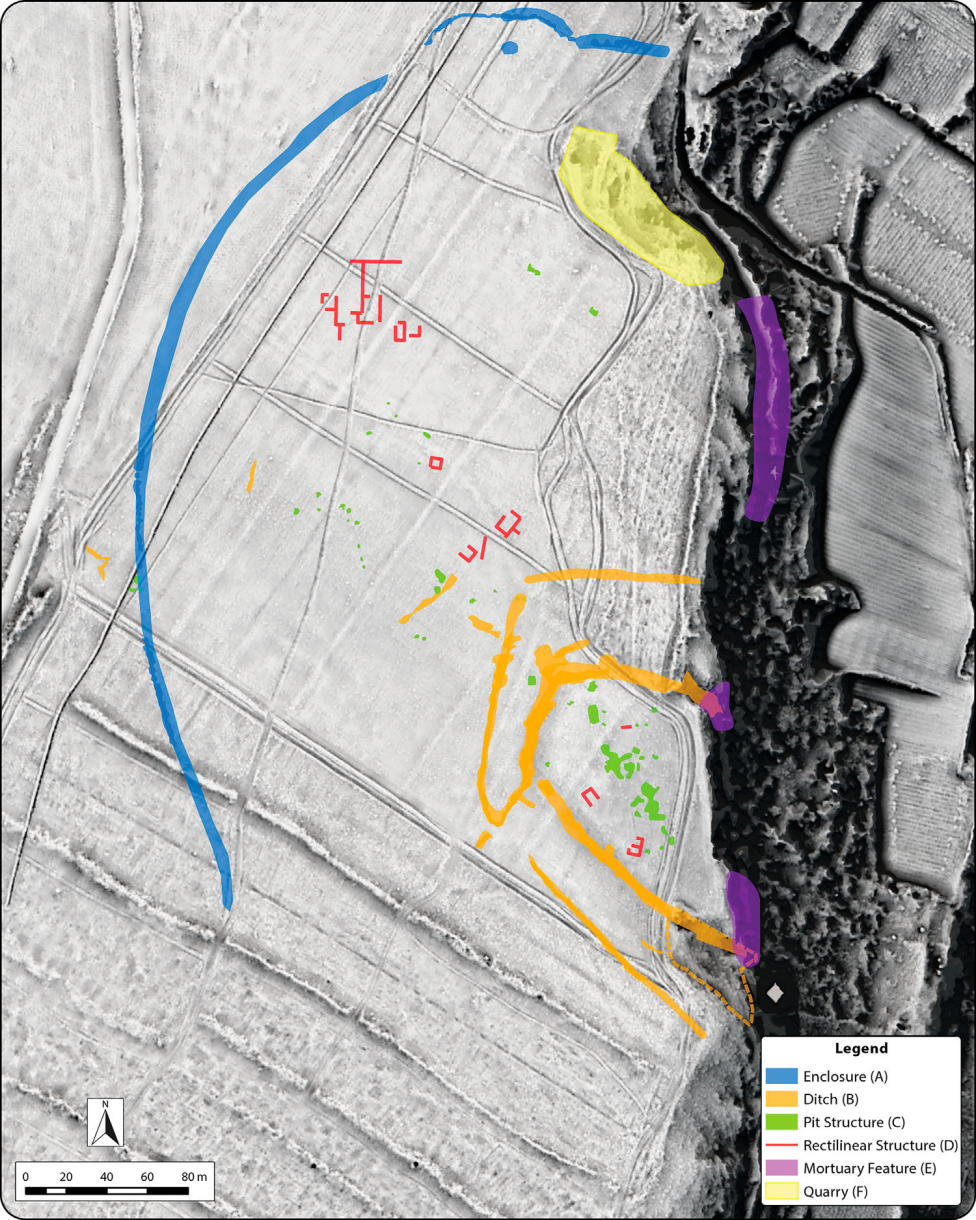
Interpretation of principal pre/protohistoric features identified at Guletta. Background: 50 cm spatial resolution ALS-derived DTM visualization (Sky-view factor, local relief model and multiple hillshade).

Although disturbed by later activity, the main structures present at Guletta that we believe to be largely of pre/protohistoric origin include the outer enclosure surrounding the site, the two ditches enclosing the settlement area, the pit structures within the settlement area, and the rectilinear structure to the northwest of it. Additionally, mortuary features of various types are cut into the cliff face overlooking the gorge. Open quarries, some of which may have origins contemporary to Guletta’s occupation sequence, are located in areas where the bedrock substrate is particularly close to the ground surface (Figure 9). Although we cannot yet establish an absolute chronology for the area, we can use the information present in the prospection data to interpret the structures and establish a relative chronology to use as a working model to better understand the development of the site.

### Interpreting the various archaeological structures at Guletta

The outermost visible structure at the site is the outer enclosure (Figure 9: A). Based on the prospection data, it appears that this was constructed in a single phase and not subsequently remodeled. As we have no direct evidence for the period of construction, it could be from any time during the site’s occupation. However, the beginning of the Bronze Age in Sicily saw the construction of a few settlements with extensive enclosing fences or walls (McConnell 1995, 29–30), and the outer enclosure at Guletta could fall into this category. The enclosure could have formed a first line of defense against attackers, though compared to the inner ditches, the remnants of the outer enclosure seem ephemeral. It may also be possible that the outer enclosure was built to contain livestock or protect crops.

The dissimilar nature of the ditches surrounding the main settlement area suggests distinct periods of development (Figure 9: B). The inner ditch is of non-uniform width, and, at approximately 4–5 m, is wider with more diffuse edges, suggesting possible longer-term use and/or exposure. The wide, sloping topography of the southern edge suggests that it may have been partly built through the enhancement of natural topographic features. Where the ditch meets the edge of the gorge, it is deeply cut into the rock. Conversely, the outer ditch appears narrower, more uniform, and better defined (see Figure 5). As they both run to the edge of the Mazaro gorge, the ditches may have been constructed as demarcations of social space, for defensive purposes, hydrological functions, or a combination of these. If constructed at different points in time, they may have even served different purposes as the needs of the settlement evolved.

The inner and outer ditches connect in the southwest, where the geophysical prospection results give the impression of a large access way to the inner portion of the settlement, somewhat obscured by construction of the modern vineyard. Interpretation of the lower GPR depth slices indicates the dissimilar nature of the outer and inner ditch construction. This is particularly apparent where the structures connect. In these lower depth slices, there are additional indications that the inner ditch may have once stretched across the open area of the south entrance and that a smaller entrance may have once stood in its place (Figure 7, right inset). Our interpretation suggests that both ditches were in use concurrently for at least some part of the site’s occupation sequence: the inner ditch may have been constructed first, then later remodeled and partially infilled, with the large southern entrance being added when the outer ditch was constructed and connected to the inner ditch.

From the data available, we can consider at least three clusters of activity on the slope within the inner ditch, and some of these features may be superimposed on others. This is additional support for interpreting multiple phases of occupation, or that each area may consist of multiple, contemporary clusters of informally arranged structures (Figure 9: C). In either case, we currently see no evidence of a formally structured settlement layout (i.e. a settlement layout aligned along a gridded system). Several of these structures are in keeping with the sizes and general form of habitation structures built both in the Early/Middle Bronze Age and the Iron Age, although the information from the geophysical prospection data and associated surface artifacts is not of sufficient resolution to draw a direct connection between these examples and the features present. Given the percentages of material recovered during the surface survey, it is plausible that a number of these structures may be from the indigenous Iron Age, possibly built over or adjacent to Bronze Age structures. Regardless of their age and contemporaneity, it seems reasonable to interpret them as habitation structures and related outbuildings.

The remnants of the rectangular structure to the northwest of the ditched settlement bear morphological similarities to later rural habitation structures that begin to appear in this part of western Sicily in the later part of the 1st millennium b.c. (Figure 9: D) (e.g. Bernardini et al. 2000, 100–101; Blake and Schon 2010, 57; Cambi 2003, 148; Fentress 1998; Mosca 2016). The largest of these appears to be a multi-roomed building whose interior divisions and dimensions suggest a single household domestic habitation structure. Smaller, similarly aligned structures may be contemporary outbuildings. While influenced by the poor state of preservation, these rectilinear structures manifest in multiple datasets, are not aligned with any prior or subsequent land use, and the surface around the largest of them shows a corresponding area of differing compaction from the surrounding soil. Although the precise period of construction and possibilities of phasing are difficult to determine, it does not appear directly related to prior or subsequent land use. At the moment, it is presumed to be pre-Roman, due to the lack of later period materials recovered during the surface survey.

**Figure 10.**
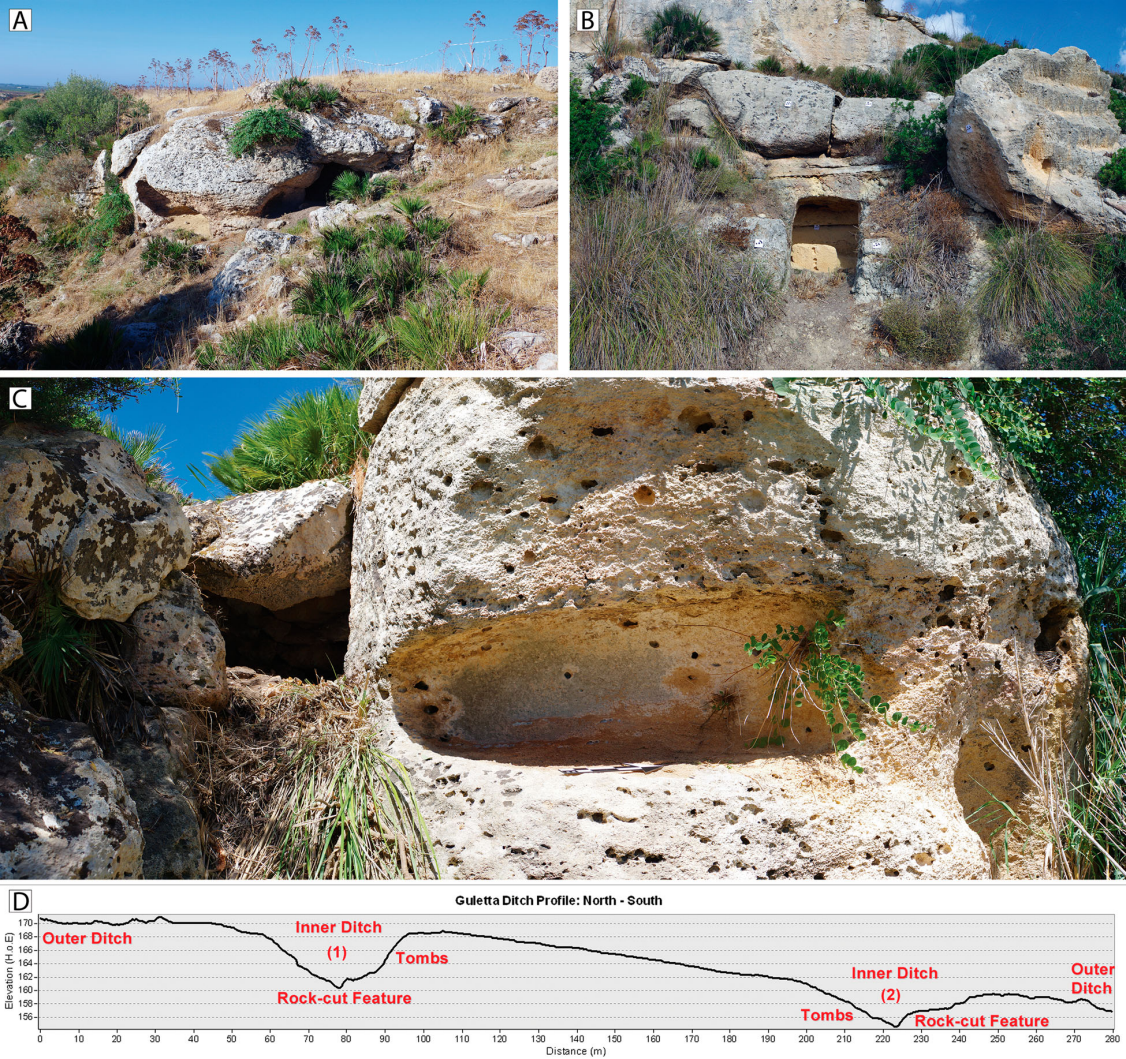
A) Tombs cut into rock outcrop in exposed portion of northern inner ditch, facing south. B) Possible Thapsos style tomb cut into exposed bedrock of cliff face, between northern edge of outer ditch and enclosure. C) Composite image of mortuary feature cut by southern inner ditch cut, facing west. D) ALS-derived profile of ditches and location of tombs. See Figure 5 for reference. Photographs: C. Sevara

The known funerary structures present near the ditched settlement are of various forms. Some of them either intersect or are intersected by the inner ditch of the settlement, providing clues to the relative stratigraphy of some of the site structures (Figure 9: E). Some tombs, located on the terraces below the northern section of the site, appear to be similar to Thapsos style tombs (Figure 10) (Leighton 1999, 166), while some appear to be of a later date. Unfortunately, these tombs are all largely empty, and we must rely mainly on morphology as an indicator of their initial construction date, a notoriously speculative exercise given the long timeframe in which some tomb types were built and reused. Nevertheless, their presence, variation, and the relative stratigraphy afforded by their intersection with other features are indicative of repeated use over a long period of time, which in turn adds to the perception of Guletta as an area of long-term habitation and use.

### Interpreting the chronology of Guletta

Given the timespan indicated by surface survey material from Guletta and the dissimilar nature of the features, it is unlikely that all features at this site represent a single construction phase. While the bulk of the material collected appears to be from the indigenous Iron Age, current knowledge about the spatial layout of contemporary indigenous settlements in western Sicily suggests that the discrete construction of a multi-ditched structure for settlement purposes would be unique. It is therefore reasonable to conclude that activity from more recent phases of occupation have partially obliterated traces from earlier periods. Furthermore, Guletta shares similarity with other MBA sites in terms of topographic position, although the ditches at Guletta are not particularly characteristic of known MBA settlement layouts in the region. Although significant material evidence for settlement at Guletta prior to the MBA is currently lacking, we can see some morphological and topographical similarities between the inner ditch at Guletta and older ditched settlements, such as those at Neolithic sites (Stintenello, Stretto Partanna, and Megara: Leighton 1999, 67–68) and Late Copper Age settlements (e.g. Heraclea Minoa: Leighton 1999, 101). In this scenario, activity at Guletta could be connected to Neolithic and Copper Age activity at the nearby settlements of Castelluccio di Mazara and Roccazzo. The presence of two quartzite axes found during surface survey hints at the possibility of activity from these periods. Additionally, the possibility of long exposure could account in part for the more diffuse nature of the inner ditch in the GPR data, lending further weight to the idea that the inner ditch may be older than most of the surface material indicates.

**Figure 11.**
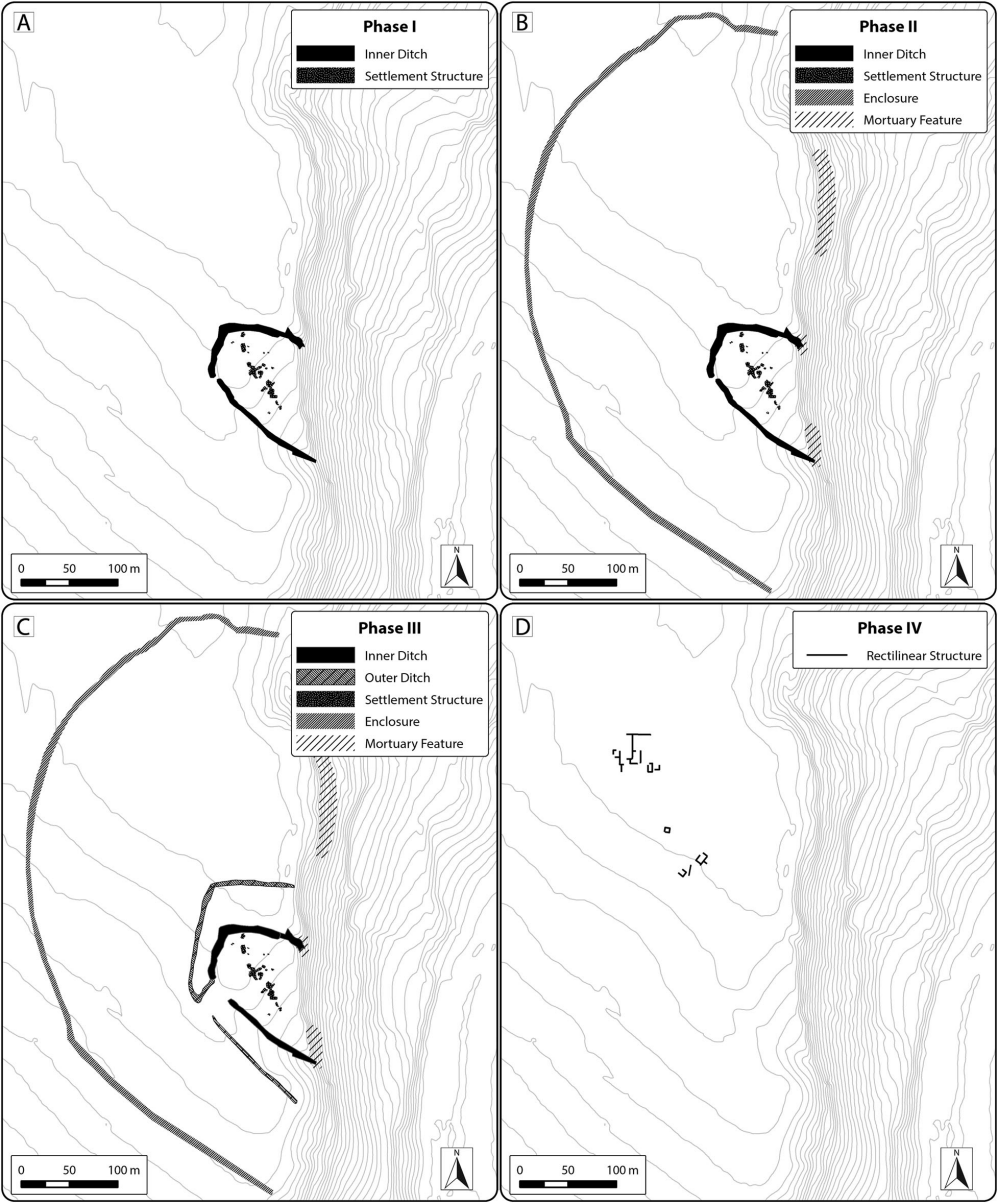
Phasing of principal pre/protohistoric structures at Guletta, based on interpretation of integrated prospection data. A) Phase I: Neolithic/Copper Age settlement layout. B) Phase 2: Middle Bronze Age Layout. C) Phase 3: Indigenous Iron Age layout. D) Phase 4: Post-6th century b.c. layout. Background: 2 m contour intervals derived from ALS DTM, smoothed to remove modern features.

Leaving aside the later period activity, our current interpretation of the pre- and protohistoric sequencing at the site indicates at least four phases of activity, possibly punctuated by periods of disuse (Figure 11). A possible scenario for Guletta’s development could be as follows: in a first phase, possibly dating to the Neolithic or Copper Age, the innermost ditch was constructed, along with settlement features (Figure 11A). The site was then abandoned and unoccupied for some time. In a second phase dating to the MBA, the inner ditch was remodeled, and additional interior structures were constructed (Figure 11B). Around or just prior to this period, mortuary features were constructed within and around the inner ditch. In a third phase, dating mainly to the indigenous Iron Age/Archaic period, the inner ditch and interior structures were again remodeled, the outer ditch was added, and mortuary features built or reused (Figure 11C). Due to the lack of overlapping features, the outermost enclosure is more difficult to place and could fit within any of these scenarios. Its state of apparent preservation leads us to believe that it may be younger rather than older, so we tentatively place it in the middle part of the sequence. In a last phase, at some point after the abandonment of the enclosed settlement, rectilinear structures were constructed (Figure 11D). Quarries may have been used throughout these periods. Subsequent to this, there is no evidence that the area covered by our survey was reoccupied for settlement purposes.

## Discussion: Establishing a Regional Context for Guletta

### Connecting nodes in later pre- and protohistoric western Sicily

Building a likely scenario for Guletta’s development is key to understanding not only the site but its place in the regional cultural landscapes of the 2nd–1st millennium b.c. Evidence for multiple phases of activity allows us to approach the question of how the site is situated within the framework of known regional activity, despite limiting chronological considerations of materials collected during the surface survey. MBA material places Guletta in the wider context of similar period sites in the region. This includes other sites along the banks of the Mazaro, such as Contrada Archi, located just a few kilometers downstream (Calafato, Tusa, and Mammina 2001, 35), as well as sites farther afield, such as Marcita, early phases of Mokarta, Monte Castellazzo di Poggioreale, Castello di Pietra, Erbe Bianchi, San Ciro, sites near Montagnola della Borrania, and pre-Phoenecian contexts on Motya (Bietti Sestieri 2015, 91; Calafato, Tusa, and Mammina 2001, 49; Ingoglia, Nicoletti, and Tusa 2012; Mannino and Spatafora 1995; Nicoletti and Tusa 2012a, 106, 113; Nigro and Spagnoli 2017; Lauro 2004, 239; Spatafora and Mannino 1992; Tusa 1999, 468, 529). In this way, Guletta may also fit in with ideas of Bronze Age settlement expansion, rather than contraction, in the west (Leighton 2005, 272). However, we cannot yet tie material from Guetta to any specific origin or cultural *facis*. We currently have no material that specifically indicates a later Bronze Age occupation at Guletta, and it may be that subsequent occupation is masking LBA activity on the site or that diagnostic materials from this period are simply difficult to identify. Alternatively, the site may have been abandoned for a period, in keeping with suggested regional trends of aggregation to larger, better-defended sites as long-distance trade contact declined (Kolb 2007, 177; Leighton 1999, 192; Tusa 1999). This possibility recommends investigating links to large inland hilltop settlements such as Mokarta, which is visible from Guletta.

**Figure 12.**
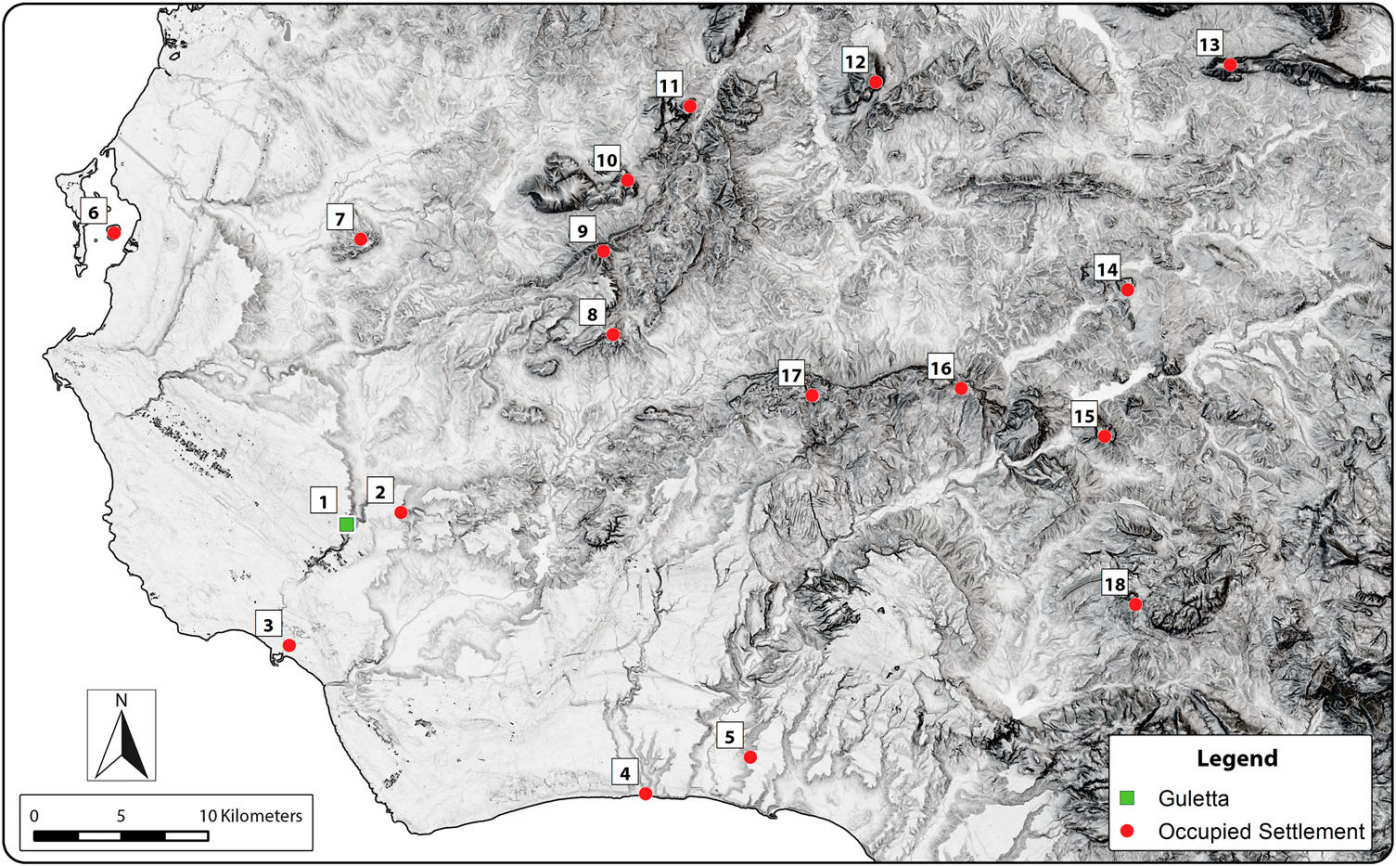
Guletta and other principal settlements occupied between the 7th–5th centuries b.c. 1. Guletta, 2. Roccazzo, 3. Mazara, 4. Selinous/Selinunte, 5. Montagnoli, 6. Motya, 7. Montagnola della Borrania, 8. Salemi, 9. Monte Polizzo, 10. Poggio Roccione, 11. Segesta, 12. Monte Bonifato, 13. Monte Iato, 14. Monte Maranfusa, 15. Entella, 16. Monte Castallazzo, 17. Monte Finistrelle, and 18. Monte Adranone. Background: DTM visualization (Sky-view factor, multiple hillshade) based on data from the Regione Siciliana geoportal.

Though present, EIA material from Guletta is of an extremely low density, and it is possible that Guletta’s occupational hiatus extended into the early part of the Iron Age, or that later activity is again masking the presence of diagnostic materials. Previous authors have noted similar chronological gaps in their survey work and have questioned whether this is due to an actual occupational hiatus, continuity in material types from the LBA, discipline-based issues surrounding the study of the period, or similar difficulties in identifying distinctive EIA material culture in survey assemblages (Leighton 2005, 280; Johns 1992; Yntema 2013, 9, 25). Nevertheless, the most prevalent material at Guletta suggests it was home to a modest indigenous 7th–5th century b.c. community that had trade contact with Phoenician and Greek interests in the west. This would make Guletta one of the westernmost of the known settlements of this type during this period (Figure 12), perhaps similar to rural settlements near Montangnola della Borrania (Falconera) and Pietra Colle (Lauro 2004, 239–40; Spanò Giammellaro, Spatafora, and van Dommelen 2008, 136, 147; Vassallo 2000, 985). Furthermore, it was occupied at the same time as larger indigenous centers further inland, such as Monte Polizzo, Monte Maranfusa, Segesta, Monte Iato, and Entella (Morris 2015, 213; Vassallo 2000, 986).

Assuming that Guletta was unoccupied or did not serve as a significant focal site during the earliest Iron Age, it may have been established in the 7th century b.c. as part of a network of contact points with new groups settling in the region. Greek settlers in the west would have benefited from local knowledge about agricultural practices and environmental exploitation, even if some of these settlers came from other parts of Sicily (De Angelis 2003b, 42). Sites such as Guletta could have served as points of contact for such purposes, as well as serving as intermediary spaces between coastal and interior zones. Guletta might also have been a prime spot for grazing animals and exploiting materials from the riverine environment of the Mazaro River, which may have been flowing in a greater capacity than it does today.

Abandonment of the settlement at Guletta may have been a part of wider regional trends during the mid-1st millennium b.c., possibly as a response to changes in social structures brought about by a growing trade economy and resulting social stratification. Environmental changes in the western Sicilian landscape, such as deforestation and the potential beginnings of ecological change in what is today the sciara may also have played a role in the changing structure and function of the site. Whatever the reason, the 6th–5th century b.c. occupants of Guletta may have joined people from other sites in the region in migrating, forcibly or otherwise, to sites that would become regional centers (Kolb 2007, 182; Morris 2015; Morris et al. 2002, 190; Vassallo 2000). There is, for instance, archaeological evidence for increased occupation levels in the fertile countryside around nearby Segesta during the second half of the 1st millennium b.c. (Bernardini et al. 2000; Spanò Giammellaro, Spatafora, and van Dommelen 2008, 132; van Dommelen and Gómez Bellard 2008, 217). We also see an intensification of Greek occupation, or at least Greek material culture, in the area between the Mazaro and Delia rivers to the south (Calafato, Tusa, and Mammina 2001, 50–51). Perhaps this abandonment and aggregation was a defensive response, or perhaps a political one, as particular native settlements strove to consolidate socioeconomic power (Morris 2015, 212). In turn, this sort of movement may be indicative of growing social stratification as people moved from a society based on agropastoralism to specializing in the production of goods, necessitating a centralized, controllable workforce focused on intensive production. Conversely, people from the settlement at Guletta may simply have been absorbed into other local communities at the edge of the Selinuntine territory*,* such as San Miceli (Mosca 2016), as activity patterns changed and resource exploitation intensified.

Around the time of the presumed abandonment of the indigenous Iron Age settlement at Guletta, Phoenician interests in western Sicily were also changing. Subsequent to the Carthaginian conquest of Selinous, an increased Punic presence can be inferred from evidence of reoccupation at sites such as Monte Polizzo and other formerly indigenous inland hilltop settlements, which seem to be reoccupied in a limited manner, possibly as military outposts (Morris et al. 2002, 191; Spanò Giammellaro, Spatafora, and van Dommelen 2008, 136; Tusa 2009, 35). If the rectilinear structure found at Guletta is indeed the remnant of later rural habitation (cf. Bernardini et al. 2000, 100–101), this may also fit in with emerging evidence of other, similar structures appearing in the landscape during the 4th century b.c. This could be related to a change in land use practices in the hinterlands of Selinunte and the eventual reorganization of agricultural practices in the coastal hinterlands of western Sicily (Blake and Schon 2010, 56; Spanò Giammellaro, Spatafora, and van Dommelen 2008, 146). Alternatively, the structure could be more elaborate than a single-family dwelling, pointing to multiple phases, earlier, and/or more complex development. Further investigation will be necessary in order to clarify this structure’s use and relationship to contemporary events in the region. Based on the artifacts recovered so far and the architectural differentiation, for the moment we can only be relatively certain that the age of this structure falls somewhere between the end of the occupation of the ditched settlement and the latter part of the first millennium b.c.

### Nodes to networks: Guletta on the border?

The cycle of material culture at Guletta may be reflective of wider trends between the MBA and Archaic/Classical periods in western Sicily. At Montagnola della Borrania, 15 km to the north of Guletta, similar patterns of occupation and abandonment seem to have occurred, particularly at site 28 (Lauro 2004, 238–242). The presence of MBA activity at Guletta, though ephemeral at present, is intriguing; further investigation may shed new light on the function of the site as a node in a regional MBA network. In contrast, the discovery of Guletta continues to push the known borders of native occupation during the indigenous Iron Age and early Archaic period in Sicily out toward the southwestern coast and establishes the first evidence of an indigenous settlement occupied into the Archaic period on the banks of the Mazaro River. Models of native settlement patterns for this period should take into account that significant events were occurring in the transition zone, and that traces of their preservation may be more difficult to identify, due to subsequent activities in these areas and the possibility of different modes of construction and occupation compared with settlements in the interior.

The sequences of abandonment and reoccupation at Guletta may be evidence of direct occupation by new cultural groups in the transition zone between coast and interior, internal socio-political struggle, or merely a change in material culture as local inhabitants engaged with new cultural and socioeconomic influences. We currently lack fine-grained contexts at the site to determine which of these is most likely and what these changes meant for local people. Nevertheless, we can infer possible connections between Guletta and other regional sites and reasons for changes in the spatial layout at Guletta based on structural evidence and the distribution and density of surface artifacts. The occupation and abandonment of Guletta fits well within general reorganizational trends in western Sicily during this period. Thus, it is likely that the occupational sequences we are seeing at Guletta reflect a wider movement from relative cultural stability to a landscape restructured by a socio-political shift from cooperation to competition.

While we currently only have enough information to speculate over such a transition, we do have evidence of a sequence of apparent habitation structures and both locally produced and imported materials that seem to come from multiple regional centers. These observations lead us to tentatively assess Iron Age Guletta as a gateway site (Rivers, Knappett, and Evans 2013, 130), a small settlement in a logistically prominent location between coast and interior, home to a mixed group of peoples living at the boundaries of three cultural entities, mediating the exchange between groups. Such a location could simultaneously fulfill needs related to spatial control, production, and flow of technologies, materials, and people until its collapse at a time of growing regional reorganization. This model could be applicable to other sites in the region, such as those at Montagnola della Borranea, and other spaces where estimated populations may not have been as large as those from contemporary interior sites but may nevertheless have had other attractive features that made them important components in regional exchange systems. Therefore, we can even visualize a network of gateway points between cultural interests, as well as coastal and interior settlements, from the MBA until their abandonment in the LIA, leveraging their positions between Phoenician activities, Greek colonial expansion, and changing indigenous interests. Investigating these concepts will be a focal point of future research.

## Conclusion

Our investigations have produced a wealth of new information at Guletta, casting it as a node of both local and regional activity in later pre- and protohistoric western Sicily. Through integrated interpretation of historic and modern remote sensing, geophysical prospection, and survey data, several previously unknown features were identified and placed chronologically between the Middle Bronze and later Archaic/Early Classical periods. In particular, prior to our investigation, settlement activity dating to the indigenous phase of the late Iron Age and Archaic period was largely unknown within our project area. Importantly, none of the features identified in the prospection data which we consider to be pre/protohistoric in origin align with any subsequent later historic period land use.

Changes in structure and material culture at the site may be related to a confluence of environmental and sociocultural changes and shifting land use practices in the middle of the 2nd and the latter part of the 1st millennium b.c. A lack of locally produced materials after the 6th–5th century b.c. and an eventual transition to the construction of a rectilinear structure could be tied to regional patterns of abandonment, aggregation, and changing land use. We see the multiple types of locally produced and imported material at the site as indicating that Guletta was, for a time, a point of interaction between different cultural groups, occupying a possible frontier zone in the transitional area between coast and interior, acting as a sociocultural/socioeconomic gateway in the region.

The comprehensive spatial nature of the information we have already obtained will allow us to target certain areas of the site and surrounding landscape for excavation and further testing to investigate specific questions related to feature dating, construction, and sequencing in a minimally destructive manner. Due to the steady amount of new material brought to the surface when firebreaks are plowed, we expect that the remnants of secure contexts persist below the surface in several places, particularly within the area bounded by the innermost ditch. Although we anticipate that our assessment of Guletta’s sequence and connections to other sites in the region will be strengthened by new information from the ongoing analysis of ceramic materials from the site, securing dates for the various features revealed by prospection would be an obvious next step in understanding site sequencing. A better understanding of the cycles and types of activity on the site will help to better situate Guletta in the context of the regional discussions about movement and interaction.

The results of our research at Guletta have provided further evidence with which to question traditional models about movement and interaction in the region. Specifically, evidence of indigenous period occupation at Guletta has redrawn the boundaries of later Iron Age settlements in the west and clearly indicates that absence of evidence was the basis of previous assumptions of regional movement in all periods. Previous intensive surveys in our project area have found little to no evidence of pre/protohistoric material beyond funerary monuments carved into the walls of the Mazaro gorge. Our findings, based on non- and minimally invasive area-based data collection approaches, indicate that evidence is certainly there. Using the information we have collected as a basis for intensive regional survey, spatial analysis, environmental testing, and further geophysical prospection, we aim to fill in more knowledge gaps about archaeological landscapes from all periods in the region.

In western Sicily and beyond, an iterative analysis of multiple types of data collected through non-invasive means, such as those employed in this study, provide us with new ways to examine past land use at multiple scales. These data can completely change our conceptions of landscapes in the past and their remains in the present. Along the Mazaro River, these data are contributing to evolving models of local and regional interaction, continuity, and change.

## Supplementary Material

Supplemental Material
