# Supplementary material for: A Landscape in Transitions: Guletta, a Multiperiod Settlement along the Mazaro River in Western Sicily
Source: J Field Archaeol. 2020 Mar 9;45(5):334–54. doi: 10.1080/00934690.2020.1734898 (PMC7446288; doi:10.1080/00934690.2020.1734898)
Supplement: Supplemental Material [file YJFA_A_1734898_SM7376.pdf]

Supplemental Material 1: Airborne Laser Scanner and RGB camera system details.

Laser Scanner: Riegl LMS-Q680i Full Waveform		RGB Camera: IGI Digicam H-39	
**Unfiltered Point-Density (per m^2)**	16	**Array Size**	39 MP
**Strip Overlap**	20%	**Detector Pitch**	6.8 µm
**Scan Angle (whole FOV)**	60°	**Image Size**	7216 × 5412 px
**Flying Height (AGL)**	511 m	**Focal Length**	50 mm
**Speed of Aircraft (TAS)**	110 knots	**Ground Sample Distance**	8 cm
**Laser Pulse Rate**	400,000 Hz	**Overlap (Side/Forward)**	30/60%
**Measurement Rate (max)**	266,000 Hz	**Exposure Time (sec)**	1/750
**Scan Lines per Second**	169	**Aperture**	*f*/5
**Laser Wavelength**	1550 nm (SWIR)	**Image Color Mode**	RGB
**Strip Adjustment/Error**	Yes, 0.0128 m (std. deviation)		
**Filtering**	Robust interpolation (OPALS)		

M = meters, FOV = field of view, AGL = above ground level, TAS = true airspeed, max = maximum, Hz = Hertz, SWIR = short-wave infrared, MP = megapixels, µm = micrometer, px = pixel, mm = millimeter, cm = centimeter, sec = seconds.

Supplemental Material 2: Parameters for geophysical prospection equipment and survey.

System	Type	Area Surveyed (ha)	Measuring Grid (m)	Resolution/ Frequency	No. Sensors/Antennas	Georeferencing
Förster Ferex	Fluxgate Magnetometer	9	0.16 × 0.5	0.1 nT, 20 meas/sec.	4 sensors	Post-processed GNSS
Sensors & Software PulseEkkoPro	Ground Penetrating Radar	2.5	0.05 × 0.25	500 MHz	3 antennas	Post-processed GNSS

Ha = hectares, m = meters, nT = nanotesla, GNSS = Global Navigation Satellite System

Sevara, C., R. B. Salisbury, M. Doneus, E. Draganits, R. Totschnig, C. Frazzetta, and S. Tusa. 2020. "A Landscape in Transitions: Guletta, a Multiperiod Settlement along the Mazaro River in Western Sicily." *Journal of Field Archaeology* 45 (5).

Supplemental Material 3: Principal prospection data sets and sources used in this study.

Data Set	Date	Time of Year	Type	Scale/ Resolution	Products	Use	Source/Provider
3187-2, 3193-8, 3198-13, 3204-19, 3205-20, 3212-27, 3213-28,3217-32, 3218-33, 3220-35, 3224-39, 3225-40, 3229-44, 3233-48, 3236-51, 3242-57, 3248-63, 3249-64, 3257-72, 3262-77, 3263-78, 3266-81, 3267-82, 3268-83, 3270-85, 3271-86, 3272-87, 3274-89, 3275-90, 3276-91, 3277-92, 3278-93, 3286-101, 3287-102, 3288-103, 3289-104, 3292-107, 3297-112, 3300-115, 3307-122	1840–1877	n.a.	Cadastral Map	1:400–1:5000	Cadastral Data	Historic Land Use Information	Archivio di Stato di Trapani http://www.archiviodistatotrapani.beniculturali.it/
257-II, 257-III, 265-I, 265-IV	1896	n.a.	Topographic Map	1:50000	Cartographic Map Mosaic	Terrain Analysis, Toponym Study, Historic Land Use Information	Istituto Geografico Militare https://www.igmi.org/

Sevara, C., R. B. Salisbury, M. Doneus, E. Draganits, R. Totschnig, C. Frazzetta, and S. Tusa. 2020. “A Landscape in Transitions: Guletta, a Multiperiod Settlement along the Mazaro River in Western Sicily.” *Journal of Field Archaeology* 45 (5).

257 II-NO, 257-II-SO, 257-III-NO, 257-II-NE, 257-III-NE, 257-III-SO, 265-IV-NO, 265-IV-NE, 265-IV-SE, 265-I-NO, 265-I-SO	1938–1941, 1969–1970	n.a.	Topographic Map	1:25000	Cartographic Map Mosaic	Terrain Analysis, Toponym Study, Historic Land Use Information	Istituto Geografico Militare
257-4-106-109	22 April 1941	Spring	Vertical B/W Photographs	1:18000	Orthomosaic, hDEM	Terrain Analysis, Land Use Information, Archaeological and Paleoenvironmental Feature Identification	Istituto Geografico Militare
257-28A-10998-11000	04 July 1955	Summer	Vertical B/W Photographs	1:30000	Orthomosaic, hDEM	Terrain Analysis, Land Use Information, Archaeological and Paleoenvironmental Feature Identification	Istituto Geografico Militare
257-IX-752-754, 257-VIII-770-774, 257-VII-788-791	09 May 1975	Spring	Vertical B/W Photographs	1:15000	Orthomosaic, hDEM	Terrain Analysis, Land Use Information, Archaeological and Paleoenvironmental Feature Identification	Istituto Geografico Militare
257-23-120-121, 257-24-1068-1072	24 June 1992	Summer	Vertical B/W Photographs	1:36000	Orthomosaic, hDEM	Terrain Analysis, Land Use Information, Archaeological and Paleoenvironmental Feature Identification	Istituto Geografico Militare

Sevara, C., R. B. Salisbury, M. Doneus, E. Draganits, R. Totschnig, C. Frazzetta, and S. Tusa. 2020. “A Landscape in Transitions: Guletta, a Multiperiod Settlement along the Mazaro River in Western Sicily.” *Journal of Field Archaeology* 45 (5).

2030504, 02030505	20 May 2003	Spring	Oblique Color Digital Image	12 MP	Orthomosaic	Archaeological Feature Identification	University of Vienna Aerial Archive https://luftbildarchiv.univie.ac.at/
2016 RGB Image Capture	21 February 2016	Winter	Vertical RGB Digital Image	8 cm spatial	Orthomosaic	Land Use Information, Archaeological and Paleoenvironmental Feature Identification	Airborne Technologies
2106 ALS Survey	21 February 2016	Winter	Geometric and Radiometric Data in Point Cloud Format	16 pts per m^2	DEMs (DTM, DSM, filtered for archaeological interpretation), Reflectance Map	Terrain Analysis, Land Use Information, Archaeological and Paleoenvironmental Feature Identification	Airborne Technologies
Magnetometry	April 2016	Spring	Gridded Survey	16 cm × 50 cm spatial	50 cm Spatial Resolution Magnetogram	Subsurface Feature Detection	ZAMG Archeo Prospections https://luftbildarchiv.univie.ac.at/
Ground Penetrating Radar	April 2016	Spring	Gridded Survey	25 × 50 cm spatial	50 cm Spatial Resolution Radargram Time Slices	Subsurface Feature Detection, Feature Depth Data	ZAMG Archeo Prospections
Surface Survey	04–07 April 2016	Spring	Intensive Linewalking Survey	2 m interpersonal distance, 100 × 100 m grid	Surface Artifact Distribution	Chronological and Tyoplogical Information	PA Prima Archeologia
Mazara del Vallo Geoportal WMS	Current	n.a.	Cadastral Map	1:5000	Cadastral Data	Modern Land Use Information	Geoportale del Commune di Mazara del Vallo http://www.comune.mazaradelvallo.sitr.it/gfmaplet/jml/

Sevara, C., R. B. Salisbury, M. Doneus, E. Draganits, R. Totschnig, C. Frazzetta, and S. Tusa. 2020. “A Landscape in Transitions: Guletta, a Multiperiod Settlement along the Mazaro River in Western Sicily.” *Journal of Field Archaeology* 45 (5).

Regione Siciliana Geoportal WMS	2007–2008	unknown	DTM derived from ALS survey	2 m spatial	DTM	Regional Topographic Data	Geoportale Regione Siciliana http://www.sitr.regione.sicilia.it/geoportale
National Geoportal WMS	1988, 1994, 2000, 2006, 2012	Various	B/W and Color Vertical Photography, Land Use Data	1 m–50 cm spatial	Orthomosaic (VIA WMS)	Historic Land Use Information	Geoportale Nazionale http://www.sitr.regione.sicilia.it/geoportale
Google Earth Pro	2004, 2006, 2010, 2011, 2013, 2015, 2016, 2017	Spring–Fall	Vertical Color Imagery	1 m–50 cm spatial	Orthomosaic (VIA WMS)	Historic Land Use Information	Google Earth https://www.google.com/earth/

n.a.= not applicable, ALS = airborne laser scanning, hDEM = historic Digital Elevation Model, DSM = digital surface model, DTM = digital terrain model, MP = megapixel, cm = centimeter, m = meter, pts = points

Sevara, C., R. B. Salisbury, M. Doneus, E. Draganits, R. Totschnig, C. Frazzetta, and S. Tusa. 2020. “A Landscape in Transitions: Guletta, a Multiperiod Settlement along the Mazaro River in Western Sicily.” *Journal of Field Archaeology* 45 (5).
